# A Phase 2 Randomized Trial Evaluating the Antiviral Activity and Safety of the Direct-Acting Antiviral Bemnifosbuvir in Ambulatory Patients with Mild or Moderate COVID-19 (MOONSONG Study)

**DOI:** 10.1128/spectrum.00077-23

**Published:** 2023-06-20

**Authors:** Marta Boffito, Eamon Dolan, Karishma Singh, William Holmes, Steffen Wildum, Arantxa Horga, Keith Pietropaolo, Xiao-Jian Zhou, Barry Clinch, Neil Collinson, Vincent Ukachukwu

**Affiliations:** a Chelsea and Westminster Hospital NHS Foundation Trust London, United Kingdom; b Imperial College London, London, United Kingdom; c Connolly Hospital, Dublin, Ireland; d Tower Family Healthcare, Bury, United Kingdom; e Roche Products Ltd., Welwyn Garden City, United Kingdom; f Roche Pharma, Research and Early Development, Roche Innovation Center Basel, Basel, Switzerland; g Atea Pharmaceuticals, Boston, Massachusetts, USA; National Institute of Allergy and Infectious Diseases

**Keywords:** AT-527, COVID-19, SARS-CoV-2, bemnifosbuvir, direct-acting antiviral

## Abstract

Bemnifosbuvir is an oral antiviral drug with a dual mechanism of action targeting viral RNA polymerase, with *in vitro* activity against SARS-CoV-2. We conducted a phase 2, double-blind study evaluating the antiviral activity, safety, efficacy, and pharmacokinetics of bemnifosbuvir in ambulatory patients with mild/moderate COVID-19. Patients were randomized 1:1 to bemnifosbuvir 550 mg or placebo (cohort A) and 3:1 to bemnifosbuvir 1,100 mg or placebo (cohort B); all doses were given twice daily for 5 days. The primary endpoint was a change from baseline in the amount of nasopharyngeal SARS-CoV-2 viral RNA by reverse transcription PCR (RT-PCR). The modified intent-to-treat infected population comprised 100 patients (bemnifosbuvir 550 mg, *n *= 30; bemnifosbuvir 1,100 mg, *n *= 30; cohort A placebo, *n *= 30; cohort B placebo, *n *= 10). The primary endpoint was not met: the difference in viral RNA adjusted means at day 7 was −0.25 log_10_ copies/mL between bemnifosbuvir 550 mg and cohort A placebo (80% confidence interval [CI], −0.66 to 0.16; *P* = 0.4260), and −0.08 log_10_ copies/mL between bemnifosbuvir 1,100 mg and pooled placebo (80% CI, −0.48 to 0.33; *P* = 0.8083). Bemnifosbuvir 550 mg was well tolerated. Incidence of nausea and vomiting was higher with bemnifosbuvir 1,100 mg (10.0% and 16.7% of patients, respectively) than pooled placebo (2.5% nausea, 2.5% vomiting). In the primary analysis, bemnifosbuvir did not show meaningful antiviral activity on nasopharyngeal viral load as measured by RT-PCR compared with placebo in patients with mild/moderate COVID-19. The trial is registered at ClinicalTrials.gov under registration number NCT04709835.

**IMPORTANCE** COVID-19 continues to be a major global public health challenge, and there remains a need for effective and convenient direct-acting antivirals that can be administered outside health care settings. Bemnifosbuvir is an oral antiviral with a dual mechanism of action and potent *in vitro* activity against SARS-CoV-2. In this study, we evaluated the antiviral activity, safety, efficacy, and pharmacokinetics of bemnifosbuvir in ambulatory patients with mild/moderate COVID-19. In the primary analysis, bemnifosbuvir did not show meaningful antiviral activity compared with placebo as assessed by nasopharyngeal viral loads. The negative predictive value of nasopharyngeal viral load reduction for clinical outcomes in COVID-19 is currently unclear, and further evaluation of bemnifosbuvir for COVID-19 may be warranted despite the findings observed in this study.

## INTRODUCTION

Coronavirus disease 2019 (COVID-19) remains a major global public health challenge, with over 620 million cases reported and more than 6.5 million deaths worldwide as of October 2022 (1). Most individuals (≥90%) infected with severe acute respiratory syndrome coronavirus 2 (SARS-CoV-2), which causes COVID-19, are asymptomatic or have mild to moderate influenza-like symptoms, but approximately 5 to 10% of all patients are hospitalized, with rates varying by viral variant (2–4).

Several COVID-19 vaccines, neutralizing monoclonal antibody therapies, and direct-acting antivirals (DAAs) are now available (5–9). Vaccinations have been effective at reducing the risk of hospitalization and death from severe COVID-19, but breakthrough infections still occur (10, 11). There are also populations for whom vaccinations are contraindicated or do not provide adequate protection, as well those who are hesitant or refuse to receive vaccinations; treatment options are therefore needed for these groups (12–14). Monoclonal antibody treatments require administration in a health care setting, leading to a demand for treatments that are orally available (7, 15). Moreover, due to the emergence of variants with potential resistance to vaccines and/or monoclonal antibodies (16, 17), antiviral treatments targeting conserved regions of the SARS-CoV-2 virus other than the spike protein will be valuable additions to the current treatment options.

There are currently 2 DAAs approved in Europe and the United States for treating adult ambulatory patients with COVID-19 at increased risk for progressing to severe COVID-19: nirmatrelvir, a protease inhibitor administered orally with ritonavir (8, 18), and remdesivir, an RNA-dependent RNA polymerase (RdRp) inhibitor requiring intravenous administration (9, 19). The requirement for ritonavir and the associated drug-drug interaction risks with nirmatrelvir/ritonavir, and the need for intravenous administration with remdesivir represent tangible limitations to the use of these agents (9, 18, 19). Another DAA, molnupiravir, is an orally administered RdRp inhibitor approved in the United Kingdom and has emergency use authorization in the United States (20, 21); however, it may be less efficacious than nirmatrelvir/ritonavir in reducing hospitalizations (22, 23). Therefore, there remains a need for additional DAAs which can be administered outside health care settings and deliver high efficacy and improved convenience.

In clinical trials of monoclonal antibodies and DAAs approved for treatment of COVID-19, the positive clinical outcomes observed have typically correlated with viral load reduction in the nasopharyngeal space, and indeed, nasopharyngeal viral load reduction has been widely used as a measure of antiviral activity in the development of antivirals for respiratory viral infections (23–29). However, its negative predictive value for clinical outcomes in COVID-19 is uncertain, as the nucleoside/nucleotide analogue, remdesivir, has demonstrated positive outcomes in clinical trials despite a lack of effect on nasopharyngeal viral load reduction (30).

Bemnifosbuvir (AT-527), the hemisulfate salt of AT-511, is an orally administered guanosine nucleotide analog double prodrug with potent *in vitro* activity against SARS-CoV-2 (31). Phase 1 and preclinical tissue distribution data predicted that bemnifosbuvir 550 mg twice daily for 5 days would achieve an effective lung concentration of the active form, AT-9010, based on *in vitro* 90% effective concentration (EC_90_) data (31). A bronchoalveolar lavage study in healthy participants further confirmed the achievement of antivirally effective levels of the drug in the lungs (32). Bemnifosbuvir has a dual mechanism of action, inhibiting viral RNA replication by blocking *de novo* initiation via the RdRp and blocking priming mediated by the Nidovirus RdRp-associated nucleotidyltransferase (NiRAN) (33).

Here, we report the antiviral activity, safety, efficacy, and pharmacokinetic results from a phase 2 study of bemnifosbuvir versus placebo in ambulatory patients with mild or moderate COVID-19.

## RESULTS

### Demographics and baseline characteristics.

**FIG 1:**
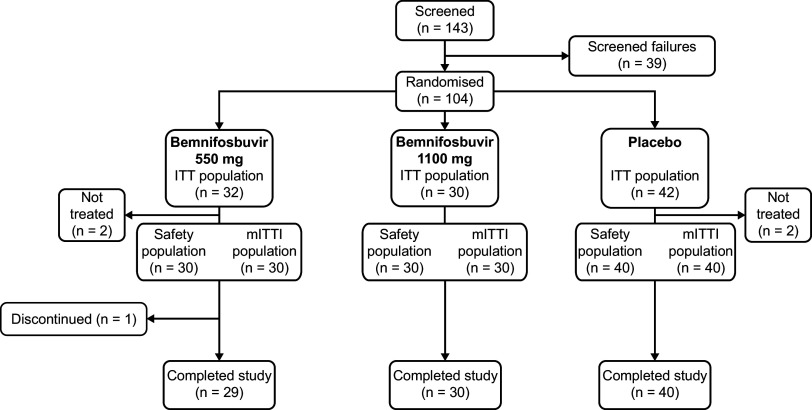
Patient disposition. The mITTI population is defined as all patients that received at least 1 dose of study treatment and had at least 1 positive SARS-CoV-2 RT-PCR test result during the study. The safety population was defined as all patients who received at least 1 dose of study treatment. ITT, intent-to-treat population; mITTI, modified intent-to-treat infected population; RT-PCR, reverse transcription PCR; SARS-CoV-2, severe acute respiratory syndrome coronavirus 2.

The study was conducted between February 2021 and October 2021 and closed after completion of recruitment of cohorts A and B, as additional cohorts were not required. Of 143 patients screened, 104 were randomized and 100 were included in the mITTI population (bemnifosbuvir 550 mg, *n *= 30; bemnifosbuvir 1,100 mg, *n *= 30; pooled placebo, *n *= 40; Fig. 1).

Demographics and baseline characteristics are presented in Table 1. Key differences between arms were observed in age, proportion of patients with underlying health conditions, baseline viral load, serostatus, vaccination status, and viral variants.

**TABLE 1:** Demographics and baseline characteristics (pooled placebo, cohorts A, B and cohorts A, B placebo)^*a*^

Demographic trait	Bemnifosbuvir 550 mg (*n *= 30)	Bemnifosbuvir 1,100 mg (*n *= 30)	Pooled placebo (*n *= 40)	Cohort A placebo (*n *= 30)	Cohort B placebo (*n *= 10)
Age in yrs					
Mean (SD)	32.7 (10.9)	40.3 (15.6)	36.8 (13.2)	34.3 (12.0)	44.4 (14.4)
Median (range)	30.5 (19–56)	39.0 (19–68)	36.5 (18–62)	34.0 (18–54)	49.0 (20–62)
Sex, *n* (%)					
Male	15 (50.0)	13 (43.3)	18 (45.0)	14 (46.7)	4 (40.0)
Female	15 (50.0)	17 (56.7)	22 (55.0)	16 (53.3)	6 (60.0)
Race, *n* (%)					
American Indian or Alaska native	1 (3.3)	0	0	0	0
Asian	0	1 (3.3)	0	0	0
Black/African American	0	1 (3.3)	2 (5.0)	0	2 (20.0)
Native Hawaiian or Pacific Islander	0	0	0	0	0
White	29 (96.7)	28 (93.3)	38 (95.0)	30 (100)	8 (80.0)
Country, *n* (%)					
Canada	0	0	1 (2.5)	0	1 (10.0)
Spain	6 (20.0)	3 (10.0)	6 (15.0)	4 (13.3)	2 (20.0)
United Kingdom	23 (76.7)	22 (73.3)	31 (77.5)	26 (86.7)	5 (50.0)
Greece	0	3 (10.0)	1 (2.5)	0	1 (10.0)
Ireland	1 (3.3)	0	0	0	0
Latvia	0	2 (6.7)	1 (2.5)	0	1 (10.0)
Days from symptom onset to study treatment					
≤3	7 (23.3)	8 (26.7)	12 (30.0)	9 (30.0)	3 (30.0)
>3	23 (76.7)	22 (73.3)	28 (70.0)	21 (70.0)	7 (70.0)
Baseline symptom severity, *n* (%)					
0: None	0	2 (7.1)	1 (2.5)	1 (3.3)	0
1: Mild	19 (65.5)	19 (67.9)	22 (55.0)	15 (50.0)	7 (70.0)
2: Moderate	10 (34.5)	7 (25.0)	17 (42.5)	14 (46.7)	3 (30.0)
3: Severe	0	0	0	0	0
Underlying conditions, *n* (%)					
Yes	7 (23.3)	14 (46.7)	18 (45.0)	11 (36.7)	7 (70.0)
Underlying conditions, *n* (%)					
Age >50 yrs	2 (6.7)	9 (30.0)	7 (17.5)	3 (10.0)	4 (40.0)
Obesity (BMI >30)	6 (20.0)	5 (16.7)	11 (27.5)	9 (30.0)	2 (20.0)
Cardiovascular disease^*b*^	0	6 (20.0)	4 (10.0)	1 (3.3)	3 (30.0)
Chronic lung disease^*c*^	2 (6.7)	2 (6.7)	1 (2.5)	0	1 (10.0)
Chronic metabolic disease^*d*^	0	1 (3.3)	2 (5.0)	2 (6.7)	0
Chronic kidney disease	0	0	0	0	0
Chronic liver disease	0	0	1 (2.5)	0	1 (10.0)
Immunocompromised	0	0	0	0	0
Baseline viral load (log_10_ copies/mL)					
Mean (SD)	6.98 (1.10)	5.94 (1.46)	6.24 (1.40)	6.46 (1.23)	5.57 (1.70)
Median (range)	7.22 (4.4–8.6)	6.16 (2.1–8.6)	6.45 (3.0–8.7)	6.47 (3.6–8.7)	5.84 (3.0–8.0)
COVID-19 vaccination status, *n* (%)					
Yes	5 (16.7)	13 (43.3)	12 (30.0)	8 (26.7)	4 (40.0)
Baseline infectious titer, *n* (%)					
Positive	24 (80.0)	19 (63.3)	28 (70.0)	22 (73.3)	6 (60.0)
Negative	6 (20.0)	11 (36.7)	12 (30.0)	8 (26.7)	4 (40.0)
Baseline serostatus, *n* (%)					
Positive	16 (53.3)	26 (86.7)	29 (72.5)	21 (70.0)	8 (80.0)
Negative	14 (46.7)	4 (13.3)	11 (27.5)	9 (30.0)	2 (20.0)
SARS-CoV-2 variant, *n* (%)					
Alpha	5 (16.7)	0	8 (22.2)	8 (27.6)	0
Delta	25 (83.3)	27 (100)	26 (72.2)	20 (69.0)	6 (85.7)
Non-variant of concern	0	0	2 (5.6)	1 (3.4)	1 (14.3)

a BMI, body mass index; SD, standard deviation.

b Including hypertension.

c Including moderate to severe asthma.

d Including diabetes.

### Virologic endpoints.

**FIG 2:**
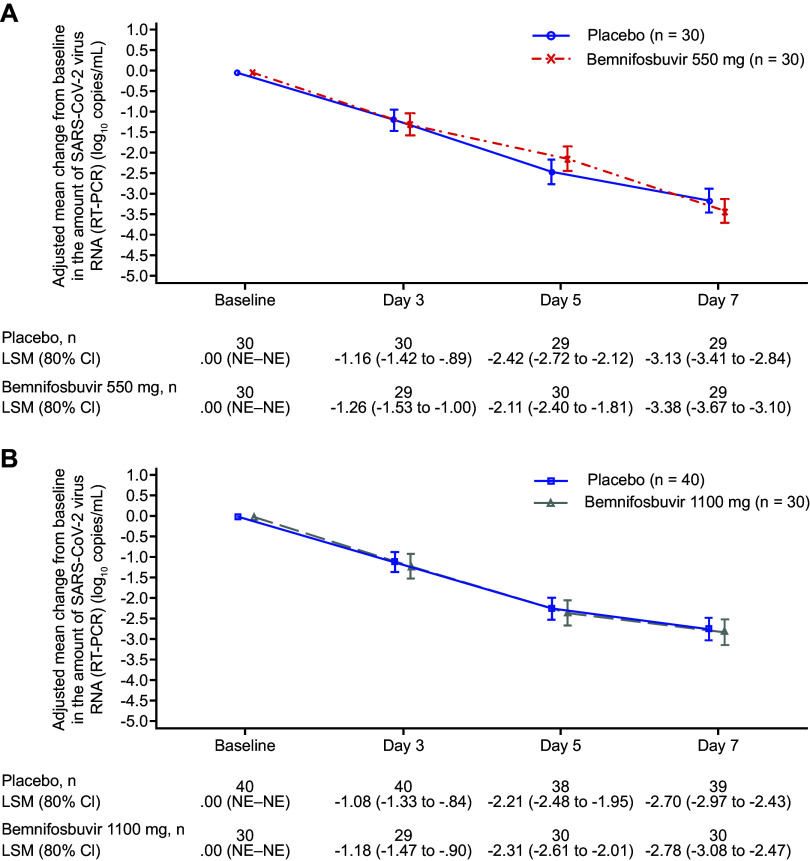
Adjusted change from baseline in the amount of SARS-CoV-2 virus RNA by RT-PCR. (A) Cohort A and cohort A placebo. (B) Cohort B and pooled placebo (modified intent-to-treat infected population). CI, confidence interval; LSM, least-squares mean; NE, not evaluable; RT-PCR, reverse transcription PCR; SARS-CoV-2, severe acute respiratory syndrome coronavirus 2.

The primary endpoint was not met; there was no significant difference in change from baseline in amount of SARS-CoV-2 RNA by reverse transcription PCR (RT-PCR) for cohort A bemnifosbuvir 550 mg versus cohort A placebo or cohort B bemnifosbuvir 1,100 mg versus pooled placebo at day 3, 5, or 7 (Table 2). The difference in adjusted means at day 7 was −0.25 log_10_ copies/mL between cohort A bemnifosbuvir 550 mg and cohort A placebo (80% confidence interval [CI], −0.66 to 0.16; *P* = 0.4260; Fig. 2A). The difference in adjusted means at day 7 between cohort B bemnifosbuvir 1,100 mg and pooled placebo was −0.08 log_10_ copies/mL (80% CI, −0.48 to 0.33; *P* = 0.8083; Fig. 2B).

**TABLE 2:** Adjusted change from baseline and difference in change from baseline in amount of SARS-CoV-2 virus RNA as measured by RT-PCR at specified time points (cohort A versus cohort A placebo and cohort B versus pooled placebo)

Change from baseline in amount of SARS-CoV-2 virus RNA^*a*^	Bemnifosbuvir 550 mg	Cohort A placebo	Bemnifosbuvir 1,100 mg	Pooled placebo
Day 3, *n*	29	30	29	40
Adjusted mean (SE)	–1.26 (0.206)	–1.16 (0.203)	–1.18 (0.223)	–1.08 (0.190)
Difference in adjusted means (SE)	–0.11 (0.292)		–0.10 (0.294)	
80% CI for difference in adjusted means	–0.49 to 0.27		–0.48 to 0.28	
Two-sided *P* value	0.7144		0.7351	
Day 5, *n*	30	29	30	38
Adjusted mean (SE)	–2.11 (0.226)	–2.42 (0.230)	–2.31 (0.231)	–2.21 (0.205)
Difference in adjusted means (SE)	0.32 (0.327)		–0.10 (0.310)	
80% CI for difference in adjusted means	–0.11 to 0.74		–0.50 to 0.30	
Two-sided *P* value	0.3373		0.7524	
Day 7, *n*	29	29	30	39
Adjusted mean (SE)	–3.38 (0.220)	–3.13 (0.220)	–2.78 (0.236)	–2.70 (0.207)
Difference in adjusted means (SE)	–0.25 (0.315)		–0.08 (0.314)	
80% CI for difference in adjusted means	–0.66–0.16		–0.48–0.33	
Two-sided *P* value	0.4260		0.8083	

a SE, standard error.

**FIG 3:**
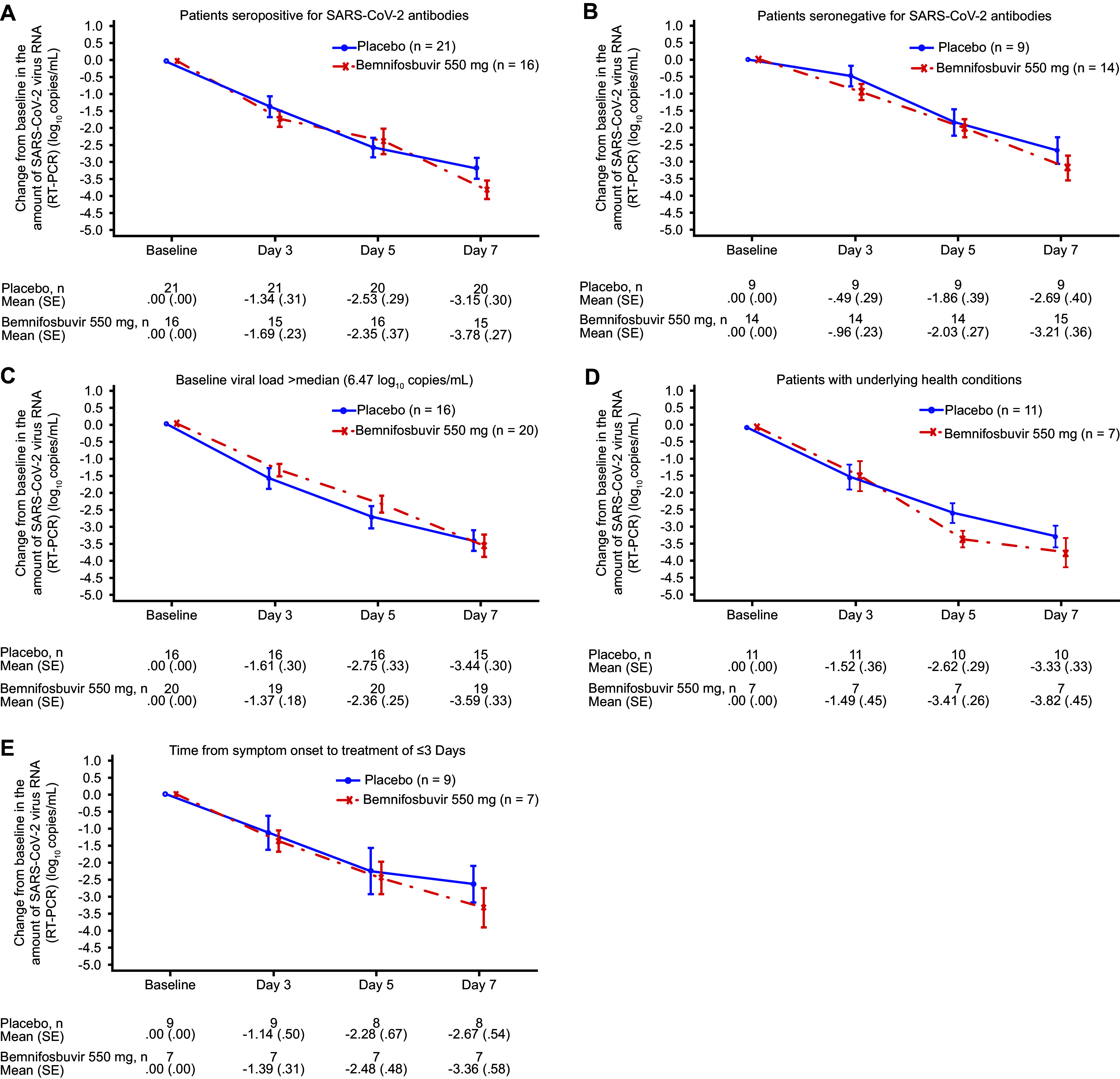
Change from baseline in the amount of SARS-CoV-2 virus RNA by RT-PCR at specified time points in key subgroups (cohort A versus cohort A placebo). RT-PCR, reverse transcription PCR; SARS-CoV-2, severe acute respiratory syndrome coronavirus 2; SE, standard error.

**FIG 4:**
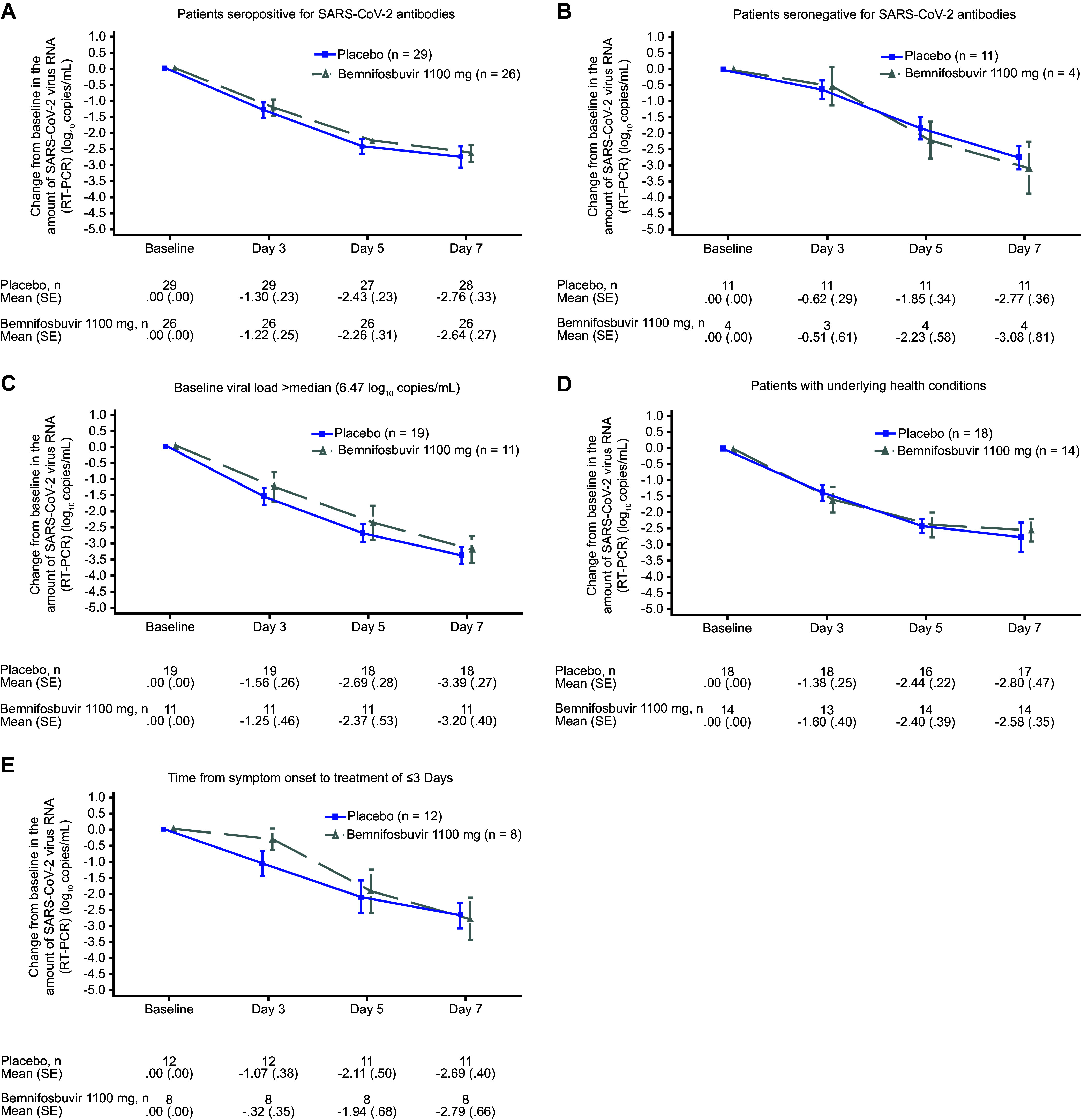
Change from baseline in the amount of SARS-CoV-2 virus RNA by RT-PCR at specified time points plot in key subgroups (cohort B versus pooled placebo). RT-PCR, reverse transcription PCR; SARS-CoV-2, severe acute respiratory syndrome coronavirus 2; SE, standard error.

Analyses in key subgroups for both cohorts were consistent with the primary analysis; subgroups analyzed included patients who were seropositive or seronegative for SARS-CoV-2 antibodies at baseline, had at least 1 underlying health condition (per Table 1), had high baseline viral load, and had time from symptom onset to treatment of ≤3 days (Fig. 3 and 4; see Fig. S1 in the supplemental material). Exploratory subgroup analyses of vaccinated and unvaccinated patients in both cohorts were also consistent with the primary analysis; no meaningful difference in the change from baseline in SARS-CoV-2 RNA measured by RT-PCR was observed for either the 550 mg or the 1,100 mg arm versus placebo in vaccinated or unvaccinated groups.

**FIG 5:**
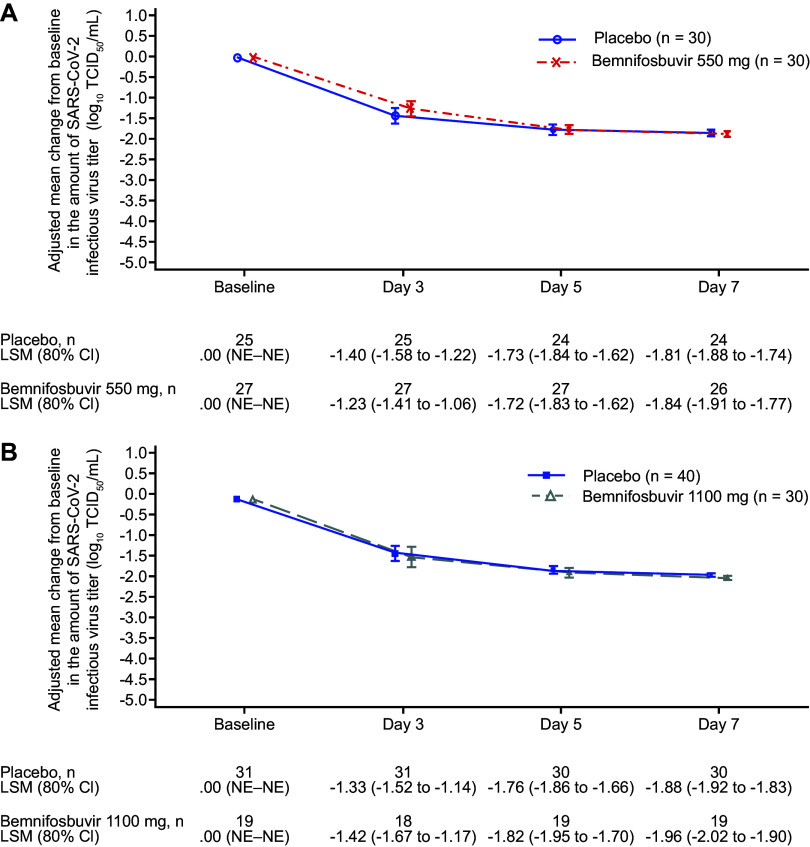
Exploratory analysis: adjusted change from baseline in the amount of SARS-CoV-2 infectious virus titer at specified time points. (A) Cohort A versus cohort A placebo. (B) Cohort B versus pooled placebo. Only patients that had at least one positive test result during the study were included. CI, confidence interval; LSM, least-squares mean; NE, not evaluable; SARS-CoV-2, severe acute respiratory syndrome coronavirus 2.

Exploratory analyses also found no meaningful difference between cohort A and cohort A placebo or between cohort B and pooled placebo in adjusted change from baseline in infectious virus titer (log_10_ 50% tissue culture infective dose (TCID_50_)/mL; Fig. 5). Subgroup analyses of infectious virus titers (log_10_ TCID_50_/mL) were consistent with the primary analysis (Fig. S2 and S3).

**FIG 6:**
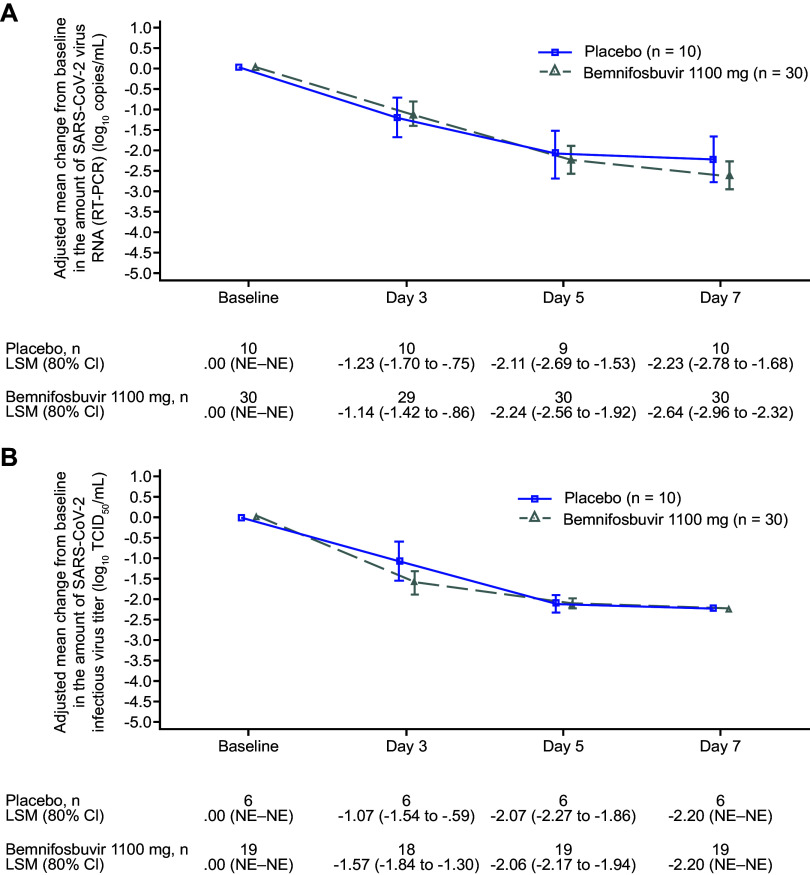
(A and B) *Post hoc* analysis: adjusted change from baseline in the amount of SARS-CoV-2 (A) virus RNA by RT-PCR and (B) infectious virus titer at specified time points; cohort B versus cohort B placebo. (B) Only patients that had at least one positive test result during the study were included. CI, confidence interval; LSM, least-squares mean; NE, not evaluable; RT-PCR, reverse transcription PCR; SARS-CoV-2, severe acute respiratory syndrome coronavirus 2.

In a *post hoc* analysis of adjusted change from baseline in amount of SARS-CoV-2 RNA by RT-PCR in the cohort B versus concurrently enrolled cohort B placebo (*n *= 10), conducted to account for differences in baseline characteristics between cohort A and cohort B placebo groups, no significant difference was observed between arms at days 3, 5, and 7 (Table 3; Fig. 6A), and no meaningful difference in change from baseline was observed in key subgroups (Fig. S4). The same analysis based on infectious virus titer showed a numerically larger adjusted mean reduction from baseline in infectious virus titer (log_10_ TCID_50_/mL) in the bemnifosbuvir 1,100 mg arm versus placebo at day 3 (Fig. 6B). Analyses of adjusted change from baseline in infectious virus titer in cohort B versus cohort B placebo from key subgroups are reported in Fig. S5.

**TABLE 3:** *Post hoc* analysis of change from baseline and difference in change from baseline in amount of SARS-CoV-2 virus RNA as measured by RT-PCR at specified time points (cohort B versus cohort B placebo)

Change from baseline in amount of SARS-CoV-2 virus RNA^*a*^	Bemnifosbuvir 1,100 mg (*n *= 30)	Cohort B placebo (*n *= 10)
Day 3, *n*	29	10
Adjusted mean (SE)	–1.14 (0.215)	–1.23 (0.367)
Difference in adjusted means (SE)	0.09 (0.426)	
80% CI for difference in adjusted means	–0.47–0.64	
Two-sided *P* value	0.8351	
Day 5, *n*	30	9
Adjusted mean (SE)	–2.24 (0.245)	–2.11 (0.447)
Difference in adjusted means (SE)	–0.13 (0.510)	
80% CI for difference in adjusted means	–0.80 to 0.53	
Two-sided *P* value	0.7988	
Day 7, *n*	30	10
Adjusted mean (SE)	–2.64 (0.244)	–2.23 (0.423)
Difference in adjusted means (SE)	–0.41 (0.489)	
80% CI for difference in adjusted means	–1.05 to 0.23	
Two-sided *P* value	0.4078	

a SE, standard error.

The proportion of patients positive for SARS-CoV-2 RNA by RT-PCR in the cohort A and B bemnifosbuvir arms was similar to that in the pooled placebo group at days 3, 5, and 7 (Table S1). In an exploratory analysis of the proportion of patients with positive SARS-CoV-2 infectious virus titer, no meaningful difference was observed between arms (Table S2); most patients were negative by day 5.

There was no meaningful difference in time to cessation of viral shedding (TCVS; based on viral RNA) for cohorts A or B versus pooled placebo (hazard ratio, 0.96 for the bemnifosbuvir 550 mg arm, 80% CI, 0.53 to 1.74; 1.33 for the bemnifosbuvir 1,100 mg arm, 80% CI, 0.76 to 2.32; Fig. S6). The median TCVS was not evaluable. There was no meaningful difference in time to sustained nondetectable virus RNA between cohorts A and B versus pooled placebo (Fig. S7). Analyses of the area under the curve of the amount of SARS-CoV-2 virus RNA by RT-PCR are reported in Table S3.

Supplementary analyses of the primary endpoint are reported in (Tables S4 to S6).

### Efficacy.

No difference was observed in time to alleviation or improvement of COVID-19 symptoms maintained for 21.5 h between the arms; median time to alleviation or improvement of symptoms was 57.2 h (80% CI, 24.6 to 80.4), 56.4 h (80% CI, 33.9 to 79.0), and 43.4 h (80% CI, 34.3 to 46.3) in the bemnifosbuvir 550 mg, bemnifosbuvir 1,100 mg, and pooled placebo arms, respectively (Fig. S8).

### Safety.

The incidence of adverse events (AEs) was similar between the arms (Table 4). A higher incidence of nausea and vomiting occurred with bemnifosbuvir 1,100 mg (10.0% and 16.7% of patients, respectively) than with pooled placebo (2.5% of patients for each; Table S7). AEs leading to treatment discontinuation are reported in Table S8.

**TABLE 4:** Safety overview

Factor^*a*^	Bemnifosbuvir 550 mg (*n *= 30)	Bemnifosbuvir 1,100 mg (*n *= 30)	Pooled placebo (*n *= 40)
Patients with ≥1 AE	6 (20.0)	10 (33.3)	11 (27.5)
Total no. of AEs	21	20	15
Deaths	0	0	0
Patients with ≥1			
AE with fatal outcome	0	0	0
Serious AE	1 (3.3)	1 (3.3)	1 (2.5)
Serious AE leading to treatment withdrawal	0	0	0
Serious AE leading to withdrawal from study	0	0	0
Related serious AE	0	0	0
AE leading to treatment withdrawal	0	5 (16.7)	1 (2.5)
AE leading to withdrawal from study	0	0	0
Related AE	2 (6.7)	5 (16.7)	5 (12.5)
Related AE leading to treatment withdrawal	0	5 (16.7)	1 (2.5)
Related AE leading to withdrawal from study	0	0	0
Grade ≥3 AE	1 (3.3)	1 (3.3)	1 (2.5)
AE of special interest	0	0	0

a AE, adverse event.

### Pharmacokinetics.

At day 5, steady-state mean trough plasma concentrations of AT-273, a plasma metabolite representative of intracellular AT-9010, were slightly below and 1.8-fold above (bemnifosbuvir 550 mg and 1,100 mg arms, respectively) the EC_90_ of AT-511 in inhibiting SARS-CoV-2 replication *in vitro*. Mean pre- and postdose plasma concentrations of bemnifosbuvir metabolites indicated that dose proportionality was achieved between 550 mg and 1,100 mg. Additional results are reported in Fig. S9 to S12.

## DISCUSSION

In this phase 2, randomized, placebo-controlled study of the oral antiviral bemnifosbuvir in patients with mild or moderate COVID-19, bemnifosbuvir did not show any meaningful difference in antiviral activity compared with placebo. There were no statistically significant differences between treatment groups in reductions in amount of virus measured by RT-PCR or infectious virus titer across the doses and subgroups analyzed. Bemnifosbuvir was well tolerated at the 550-mg dose; an increase in nausea and vomiting was observed with the 1,100-mg dose.

Previous preclinical and phase I studies have predicted that bemnifosbuvir 550 mg twice daily would achieve an effective lung concentration (31). Further, a bronchoalveolar lavage study in two cohorts of eight individuals confirmed that bemnifosbuvir 550 mg twice daily for 2.5 days achieved an antivirally relevant exposure in the lungs 4 h after the final dose (32). These results support the conclusion that a lack of antiviral activity observed in the MOONSONG study was not due to low drug exposure. Viral load reduction in the nasopharyngeal space is typically used as a measure of the antiviral activity of drugs in respiratory viral infections, including influenza and COVID-19, and traditionally provides proof of concept in early-phase trials (23–27). However, its correlation with clinical outcomes in COVID-19 appears mixed. Several antivirals for COVID-19 have shown reductions in viral load or viral clearance in the nasopharyngeal space, which were associated with clinical outcomes in trials (23, 24, 27–29). In a phase 2/3 study of high-risk, nonhospitalized adults with COVID-19, nirmatrelvir/ritonavir resulted in an 89% lower risk of progression to severe disease and was associated with a reduction in viral load by a factor of 10 compared with placebo (23). Similarly, molnupiravir was associated with a greater reduction in viral load compared with placebo in a phase 3 study of high-risk, nonhospitalized patients with COVID-19 and resulted in a 30% reduction in risk of hospitalization or death (27). In contrast, remdesivir treatment has not resulted in any viral load reduction in animal models or human clinical trials despite positive clinical outcome results in clinical trials (30). In a randomized, placebo-controlled study in outpatients with risk factors for progression to severe disease, remdesivir resulted in an 87% lower risk of hospitalization or death compared with placebo, but had no substantial effect on reduction of viral load (30). These results suggest that viral load reduction or clearance from the nasopharyngeal space has a strong positive predictive value for clinical outcomes in COVID-19, but its negative predictive value remains unclear. Considering this and the unmet need for new oral antivirals to treat COVID-19, further evaluation of clinical outcomes of bemnifosbuvir for COVID-19 may be warranted, despite the absence of viral load reduction in this study.

### Limitations.

The study was anticipated to enroll rapidly and was designed to minimize placebo enrollment in later cohorts by pooling data from placebo patients in earlier cohorts. However, slower enrollment than anticipated and rapid roll out of vaccines led to differences in key baseline characteristics such that the cohort A and B placebo groups were not fully comparable, which may have impacted the results of the cohort B primary analysis. To address this limitation, *post hoc* analyses were conducted for cohort B versus the concurrently enrolled cohort B placebo arm; however, due to the unequal randomization in cohort B, the small sample size of the cohort B placebo group was a limitation in these analyses.

### Conclusions.

In conclusion, in the primary analysis of the phase 2 MOONSONG study of patients with mild or moderate COVID-19 with or without risk factors for poor outcomes, bemnifosbuvir did not show any meaningful difference in antiviral activity as measured by RT-PCR from nasopharyngeal swabs compared with placebo. Despite this finding, further evaluation of clinical outcomes of bemnifosbuvir in the treatment of COVID-19 may be warranted given current uncertainty around the negative predictive value of viral load reduction for clinical outcomes in COVID-19.

## MATERIALS AND METHODS

### Study design and patients.

MOONSONG was a phase 2, randomized, double-blind, placebo-controlled study to assess the antiviral activity, safety, efficacy, and pharmacokinetics of different dosing regimens of bemnifosbuvir in ambulatory patients with mild or moderate COVID-19. Up to 5 cohorts evaluating selected dose regimens were planned (cohorts A to E). An interim analysis of safety and virology data was conducted in cohort A after 30 treated patients reached day 10.

Patients were recruited at 12 centers across Canada, Greece, Ireland, Latvia, Spain, and the United Kingdom. Eligible patients were aged ≥18 years with a positive SARS-CoV-2 test at screening (by RT-PCR or rapid antigen test) and mild or moderate COVID-19 symptoms (as determined by investigator), with onset ≤5 days prior to randomization. Otherwise-healthy patients and those at high risk for poor outcomes were eligible (enrollment of high-risk patients was permitted after a protocol amendment, with high risk being defined as age of >50 years, obesity, cardiovascular disease, chronic lung disease, chronic metabolic disease, chronic kidney or liver disease, or immunocompromised patients). Patients were excluded if they showed clinical signs indicating COVID-19 illness requiring hospitalization (defined as any of the following: shortness of breath at rest, respiratory rate of ≥30 breaths per minute, heart rate of ≥125 beats per minute, peripheral capillary oxygen saturation of ≤93% on room air). Full inclusion and exclusion criteria are provided in File S1.

All patients gave written informed consent to participate in the study. The study protocol was approved by an institutional review board or ethics committee. The trial was conducted according to the principles of the Declaration of Helsinki, the International Council for Harmonization Guidelines for Good Clinical Practice, and relevant country-specific laws and regulations. The study is registered at Clinicaltrials.gov (registration number NCT04709835).

### Randomization and treatment.

Patients were randomized to treatment arms by an interactive voice or web-based response system. Patients in cohort A were randomized in a 1:1 ratio to receive either bemnifosbuvir at a dose of 550 mg or placebo twice daily for 5 days, and patients in cohort B were randomized in a 3:1 ratio to receive either bemnifosbuvir 1,100 mg or placebo twice daily for 5 days.

### Study outcomes.

The primary objective of the study was to evaluate the antiviral activity of bemnifosbuvir compared with placebo through a change from baseline in the amount of nasopharyngeal SARS-CoV-2 viral RNA by RT-PCR at specified time points (days 3, 5, and 7). Secondary virologic endpoints were time to cessation of viral shedding (TCVS) measured by RT-PCR, defined as the time between the initiation of any study treatment and the first time a negative qualitative or BLQ (below the limit of quantification of 120 copies/mL) RT-PCR test result was obtained; time to sustained nondetectable virus RNA, defined as the time between initiation of any study treatment and the first time a negative qualitative or BLQ RT-PCR test result was obtained after which no positive quantitative test above or equal to the limit of quantification (LOQ) was reported; proportion of patients positive for virus RNA by RT-PCR, defined as the percentage of patients with a positive quantitative RT-PCR test result above or equal to the LOQ at each time point; and area under the curve (AUC) of virus RNA by RT-PCR. Exploratory virologic endpoints based on infectious virus measured by tissue culture infectious dose (TCID_50_) included the change from baseline in SARS-CoV-2 infectious virus titer, TCVS, AUC, and proportion of patients positive with virus at different time points. Secondary safety endpoints included the incidence and severity of adverse events (AEs). Secondary efficacy endpoints included time to alleviation or improvement of COVID-19 symptoms, maintained for 21.5 h.

### Statistical methods and sample size.

Across all 5 planned dose cohorts, 220 patients were planned for randomization. Approximately 60 patients were planned in a 1:1 ratio in cohort A to receive bemnifosbuvir or placebo. Each additional cohort was planned to enroll approximately 40 patients in a 3:1 ratio to receive bemnifosbuvir or placebo, respectively. As placebo data across cohorts were expected to be similar, analyses in cohorts B to E were planned to reuse placebo data from the preceding cohorts—hence the higher randomization ratio designed to minimize placebo enrollment into these cohorts. A sample size of 27 patients per arm was intended to ensure at least 80% power to detect a mean difference between treatment arms in change from baseline of viral RNA of between 0.7 and 1.1 log_10_ virus copies/mL measured by RT-PCR at a single time point, based on using a 2-sample *t* test for comparison of means, assuming a standard deviation of between 1.2 and 1.85 and a 1-sided 10% alpha. The sample size was adjusted to 30 patients per arm to account for an estimated 90% SARS-CoV-2 positive rate, based on a single-cohort comparison of bemnifosbuvir versus placebo.

Primary and secondary virologic outcomes were performed in the modified intent-to-treat infected (mITTI) population, defined as all randomized patients who received any amount of study drug and with at least 1 positive SARS-CoV-2 RT-PCR test at any time point during the study (regardless of the day of infection that the positive test occurred; testing was not conducted after day 7 of the study); patients were grouped according to the treatment assigned at randomization. The primary endpoint was evaluated in the bemnifosbuvir 550 mg arm versus the cohort A placebo arm and the bemnifosbuvir 1,100 mg arm versus a pooled placebo group consisting of patients from the placebo arms of both cohorts. Secondary analyses compared the bemnifosbuvir arm in each cohort with the pooled placebo group. Safety analyses were performed on the safety population, defined as all patients who received any amount of study drug; patients were grouped according to the treatment received.

The primary endpoint was analyzed using analysis of covariance with baseline virus RNA as a covariate. Further statistical methods are provided in the File S1.

### Clinical and laboratory analyses.

Nasopharyngeal swab samples were collected on days 1, 3, 5, and 7 either in the clinic or by mobile nursing, and virology assays were performed by Viroclinics Biosciences-DDL Diagnostic Laboratory (Rotterdam, Netherlands). SARS-CoV-2 viral load (RNA copies/mL) was measured by N1-gene RT-PCR as described by Lu et al. (34) with adjusted probe dyes and PCR program and an LOQ of 120 copies/mL. Infectious virus titer (TCID_50_/mL) was determined in Vero E6/TMPRSS2 cells by immunostaining using a monoclonal antibody targeting the SARS-CoV-2 nucleoprotein according to the Spearman-Karber method. Monitoring of AEs took place from screening until day 33. Patients kept a daily COVID-19 symptom diary (based on U.S. Food and Drug Administration guidance [35]) from days 1 to 28. Anti-SARS-CoV-2 spike protein antibody analysis was performed in serum samples collected on days 1 (predosing) and 7 by PPD (Highland Heights, KY, USA) using the Roche Elecsys assay. Pharmacokinetic methods are reported in the File S1.

### Data availability.

Qualified researchers may request access to individual patient-level data through the clinical study data request platform (https://vivli.org/). Further details on Roche’s criteria for eligible studies are available at https://vivli.org/members/ourmembers/. For further details on Roche’s Global Policy on the Sharing of Clinical Information and how to request access to related clinical study documents, see https://www.roche.com/research_and_development/who_we_are_how_we_work/clinical_trials/our_commitment_to_data_sharing.htm.
