# Supplementary material for: A Phase 2 Randomized Trial Evaluating the Antiviral Activity and Safety of the Direct-Acting Antiviral Bemnifosbuvir in Ambulatory Patients with Mild or Moderate COVID-19 (MOONSONG Study)
Source: Microbiol Spectr. 2023 Jun 20;11(4):e00077-23. doi: 10.1128/spectrum.00077-23 (PMC10434175; doi:10.1128/spectrum.00077-23)
Supplement: Supplemental file 1 — Supplemental material. Download spectrum.00077-23-s0001.docx, DOCX file, 2.3 MB [file spectrum.00077-23-s0001.docx]

**Supplementary Appendix**

**Supplementary Methods**

**Inclusion Criteria**

- Signed Informed Consent Form
- Age ≥18 years at time of signing Informed Consent Form
- Willing and able to comply with the study protocol and all study procedures, in the opinion of the investigator
- Positive SARS-CoV-2 diagnostic test (RT-PCR or rapid antigen test) at screening
- Had symptoms consistent with mild or moderate COVID-19, as determined by the investigator, with onset ≤5 days prior to randomization
- For women of childbearing potential: agreement to remain abstinent (refrain from heterosexual intercourse) or use adequate contraception
- Be otherwise healthy or have underlying health conditions leading to high risk for poor outcomes (defined as age >50 years, obesity, cardiovascular disease, chronic lung disease, chronic metabolic disease, chronic kidney or liver disease, or immunocompromised patients)
  - Otherwise-healthy patients only were initially eligible for enrollment; per a protocol amendment in February 2021, this was expanded to include patients with underlying health conditions leading to high risk for poor outcomes

**Exclusion Criteria**

- Clinical signs indicative of COVID-19 illness requiring hospitalization, defined as any of the following: shortness of breath at rest, respiratory rate ≥30, heart rate ≥125, peripheral capillary oxygen saturation ≤93% on room air
- Treatment with a therapeutic agent against SARS-CoV-2 including, but not limited to, other direct-acting antivirals, convalescent plasma, monoclonal antibodies against SARS-CoV-2, or intravenous immunoglobulin within 3 months or less than 5 drug-elimination half-lives (whichever is longer) prior to screening
- Requirement, in the opinion of the investigator, for any of the prohibited medications during the study
- Use of hydroxychloroquine or amiodarone within 7 days of screening
- Pregnant or breastfeeding, or intending to become pregnant during the study or within 30 days after the final dose of AT-527. Women of childbearing potential must have a negative urine pregnancy test result at screening
- Abnormal laboratory test results at screening, defined as meeting any of the following sets of criteria:
  - Alanine aminotransferase or aspartate aminotransferase >5 times upper limit of normal (ULN)
  - Total bilirubin >1.5 times ULN, unless the patient has known Gilbert disease
  - Creatinine clearance <60 mL/min
  - Total white blood cells <2,500/mm^3^ or absolute neutrophil count <800 x 109/L (1500 x L)
  - Platelet count <80 x 10^9^/L
- Clinically significant abnormal electrocardiogram, as determined by the investigator, at screening
- Planned procedure or surgery during the study
- Known allergy or hypersensitivity to study drug or drug product excipients
- Substance abuse, as determined by the investigator, within 12 months prior to screening
- Poor peripheral venous access
- Malabsorption syndrome or other condition that would interfere with enteral absorption
- Any clinically significant history of epistaxis within the last 3 months and/or history of being hospitalized due to epistaxis on any previous occasion
- History of anaphylaxis
- Any uncontrolled serious medical condition or other clinically significant abnormality in laboratory tests that, in the investigator's judgment, precludes the patient's safe participation in and completion of the study

**Additional Statistical Analysis Methods**

The primary endpoint was analyzed using analysis of covariance with baseline virus RNA as a covariate. Virologic time to event endpoints were summarized using Kaplan-Meier plots, median time to event (and 80% confidence interval [CI]), and hazard ratios from a Cox proportional hazards model. Other virologic and safety secondary endpoints were summarized using descriptive statistics. No formal statistical testing was performed for secondary efficacy endpoints; these endpoints were summarized using Kaplan–Meier plots and descriptive statistics.

**Virologic Data Imputation Rules**

The following imputation rules were applied for virology data: if the qualitative result of the SARS-CoV-2 RT-PCR test (determined using cycle threshold (Ct) values) was positive, then an associated quantitative result was also provided. If the virus RNA sample had a positive qualitative result, but a quantitative result below the limit of quantification (BLQ; 120 copies/mL), virus RNA was imputed as limit of quantification (LOQ) – 1 (119 copies/mL). Negative qualitative samples (no Ct value obtained) were imputed as LOQ/2 (60 copies/mL). Infectious viral titer samples reported as BLQ were imputed to the lower limit of quantitation minus .1 (0.9 log_10_ TCID_50_/mL). Samples reported as above limit of quantification were imputed to the upper limit of quantitation plus .1 (5.5 log_10_ TCID_50_/mL). Samples reported as negative were imputed to the limit of detection divided by 2 (.375 log_10_ TCID_50_/mL). Missing data were not imputed. Any missing data were not included in the analyses.

**Pharmacokinetic Laboratory and Statistical Analysis Methods**

Samples for pharmacokinetic (PK) analysis were taken on days 1 and 5 (pre- and post-dose), day 3 (pre-dose only), and day 7. PK samples were processed to plasma. Concentrations of AT-511 and its major metabolites, AT-551, AT-229, and AT-273, were quantified by validated liquid chromatography-tandem mass spectrometry. Descriptive summary statistics for PK concentration data were provided by cohort and visit/sampling timepoint, and include mean, standard deviation, coefficient of variation (arithmetic and geometric), median, minimum, and maximum. Concentrations below the lower limit of quantification were reported as zero.

**Supplementary Table 1. Proportion of Patients Positive for SARS-CoV-2 Virus RNA by RT-PCR (Cohorts A and B vs Pooled Placebo)**

|  | Bemnifosbuvir 550 mg  (*n* = 30) | Bemnifosbuvir 1,100 mg  (*n* = 30) | Pooled Placebo  (*n* = 40) |
| --- | --- | --- | --- |
| Baseline, n | 30 | 30 | 40 |
| Positive | 30 (100) | 29 (96.7) | 40 (100) |
| Negative | 0 | 1 (3.3) | 0 |
| Difference in proportion of positivity (80% CI) | 0.00 (NE–NE) | –3.33 (–7.01 to .34) |  |
| Day 3, n | 30 | 29 | 40 |
| Positive | 30 (100) | 27 (93.1) | 38 (95.0) |
| Negative | 0 | 2 (6.9) | 2 (5.0) |
| Difference in proportion of positivity (80% CI) | 5.00 (–.16 to 10.16) | –1.90 (–9.20 to 5.41) |  |
| Day 5, n | 30 | 30 | 38 |
| Positive | 27 (90.0) | 25 (83.3) | 32 (84.2) |
| Negative | 3 (10.0) | 5 (16.7) | 6 (15.8) |
| Difference in proportion of positivity (80% CI) | 5.79 (–4.82 to 16.40) | –.88 (–12.40 to 10.65) |  |
| Day 7, n | 29 | 30 | 39 |
| Positive | 23 (79.3) | 23 (76.7) | 30 (76.9) |
| Negative | 6 (20.7) | 7 (23.3) | 9 (23.1) |
| Difference in proportion of positivity (80% CI) | 2.39 (–10.64 to 15.42) | –.26 (–13.39 to 12.88) |  |

Abbreviations: CI, confidence interval; NE, not evaluable.

**Supplementary Table 2. Exploratory Analysis: Proportion of Patients Positive for SARS-CoV-2 by Infectious Virus Titer (TCID_50_) (Cohorts A and B vs Pooled Placebo)**

|  | Bemnifosbuvir 550 mg  (*n* = 30) | Bemnifosbuvir 1,100 mg  (*n* = 30) | Pooled Placebo  (*n* = 40) |
| --- | --- | --- | --- |
| Baseline, n | 27 | 19 | 31 |
| Positive | 24 (88.9) | 19 (100) | 28 (90.3) |
| Negative | 3 (11.1) | 0 | 3 (9.7) |
| Difference in proportion of positivity (80% CI) | –1.43 (–11.71 to 8.84) | 9.68 (.81–18.54) |  |
| Day 3, n | 27 | 18 | 31 |
| Positive | 18 (66.7) | 9 (50.0) | 14 (45.2) |
| Negative | 9 (33.3) | 9 (50.0) | 17 (54.8) |
| Difference in proportion of positivity (80% CI) | 21.51 (4.73–38.28) | 4.84 (–14.11 to 23.79) |  |
| Day 5, n | 27 | 19 | 30 |
| Positive | 8 (29.6) | 3 (15.8) | 6 (20.0) |
| Negative | 19 (70.4) | 16 (84.2) | 24 (80.0) |
| Difference in proportion of positivity (80% CI) | 9.63 (–5.00 to 24.26) | –4.21 (–18.76 to 10.34) |  |
| Day 7, n | 26 | 19 | 30 |
| Positive | 2 (7.7) | 0 | 3 (10.0) |
| Negative | 24 (92.3) | 19 (100) | 27 (90.0) |
| Difference in proportion of positivity (80% CI) | –2.31 (–12.10 to 7.48) | –10.00 (–19.01 to –.99) |  |

Only patients that had at least one positive test result during the study were included.

Abbreviation: CI, confidence interval.

**Supplementary Table 3. Area Under the Curve of the Amount of SARS-CoV-2 Virus RNA by RT-PCR (Cohorts A and B vs Pooled Placebo)**

| Area Under the Curve (log_10_ copies/mL*hour) | Bemnifosbuvir  550 mg (*n* = 30) | Bemnifosbuvir 1,100 mg (*n* = 30) | Pooled Placebo (*n* = 40) |
| --- | --- | --- | --- |
| Mean (SD) | 733.70 (146.63) | 618.62 (194.88) | 651.56 (189.10) |
| 80% CI for mean | 698.59–768.81 | 571.95–665.28 | 612.58–690.54 |
| Median | 738.06 | 594.36 | 629.27 |
| 25^th^ percentile | 645.98 | 454.04 | 533.94 |
| 75^th^ percentile | 854.96 | 772.83 | 808.63 |
| Min–max | 441.1–969.6 | 300.0–1042.0 | 292.3–1086.8 |

Abbreviations: CI, confidence interval; SD, standard deviation.

**Supplementary Table 4. Time-weighted Average Change from Baseline in Amount of SARS-CoV-2 Virus RNA by RT-PCR (Cohort A vs Cohort A Placebo)**

| Time Weighted Average Change from Baseline (log_10_ copies/mL) | Bemnifosbuvir 550 mg (*n* = 30) | Cohort A Placebo  (*n* = 30) |
| --- | --- | --- |
| Mean (SD) | –1.75 (.73) | –1.61 (.94) |
| 80% CI for mean | –1.93 to –1.58 | –1.84 to –1.39 |
| Median | –1.63 | –1.74 |
| 25^th^ percentile | –2.22 | –2.20 |
| 75^th^ percentile | –1.13 | –1.05 |
| Min–max | –3.1 to –.6 | –3.8 to –.4 |

Abbreviations: CI, confidence interval; SD, standard deviation.

**Supplementary Table 5.** **Time-weighted Average Change from Baseline in Amount of SARS-CoV-2 Virus RNA by RT-PCR (Cohort B vs Pooled Placebo)**

| Time Weighted Average Change from Baseline (log_10_ copies/mL) | Bemnifosbuvir 1,100 mg (*n* = 30) | Pooled Placebo  (*n* = 40) |
| --- | --- | --- |
| Mean (SD) | –1.60 (.94) | –1.57 (.91) |
| 80% CI for mean | –1.82 to –1.38 | –1.76 to –1.39 |
| Median | –1.92 | –1.65 |
| 25^th^ percentile | –2.24 | –2.19 |
| 75^th^ percentile | –.93 | –1.11 |
| Min–max | –3.2 to –.7 | –3.8 to –.4 |

Abbreviations: CI, confidence interval; SD, standard deviation.

**Supplementary Table 6. Time-weighted Average Change from Baseline in Amount of SARS-CoV-2 Virus RNA by RT-PCR (Cohort B vs Cohort B Placebo)**

| Time Weighted Average Change from Baseline (log_10_ copies/mL) | Bemnifosbuvir 1,100 mg (*n* = 30) | Cohort B Placebo (*n* = 10) |
| --- | --- | --- |
| Mean (SD) | –1.60 (.94) | –1.45 (.82) |
| 80% CI for mean | –1.82 to –1.38 | –1.81 to –1.09 |
| Median | –1.92 | –1.53 |
| 25^th^ percentile | –2.24 | –1.94 |
| 75^th^ percentile | –.93 | –1.29 |
| Min–max | –3.2 to –.7 | –2.4 to –.3 |

Abbreviations: CI, confidence interval; SD, standard deviation.

**Supplementary Table 7. All Adverse Events (Safety Population)**

|  | Bemnifosbuvir 550 mg  (*n* = 30) | Bemnifosbuvir 1,100 mg  (*n* = 30) | Pooled Placebo (*n* = 40) |
| --- | --- | --- | --- |
| Total patients with ≥1 AE | 6 (20.0) | 10 (33.3) | 11 (27.5) |
| Total number of AEs | 21 | 20 | 15 |
| Gastrointestinal disorders |  |  |  |
| Total patients with ≥1 AE | 2 (6.7) | 6 (20.0) | 3 (7.5) |
| Total number of AEs | 2 | 10 | 5 |
| Vomiting | 0 | 5 (16.7) | 1 (2.5) |
| Nausea | 1 (3.3) | 3 (10.0) | 1 (2.5) |
| Abdominal pain upper | 0 | 0 | 1 (2.5) |
| Abdominal tenderness | 1 (3.3) | 0 | 0 |
| Diarrhea | 0 | 1 (3.3) | 0 |
| Toothache | 0 | 0 | 1 (2.5) |
| General disorders and administration site conditions |  |  |  |
| Total patients with ≥1 AE | 3 (10.0) | 1 (3.3) | 1 (2.5) |
| Total number of AEs | 3 | 1 | 1 |
| Chest pain | 1 (3.3) | 1 (3.3) | 0 |
| Chills | 1 (3.3) | 0 | 0 |
| Mass | 0 | 0 | 1 (2.5) |
| Pyrexia | 1 (3.3) | 0 | 0 |
| Investigations |  |  |  |
| Total patients with ≥1 AE | 3 (10.0) | 0 | 2 (5.0) |
| Total number of AEs | 4 | 0 | 2 |
| Body temperature decreased | 1 (3.3) | 0 | 1 (2.5) |
| Alanine aminotransferase increased | 1 (3.3) | 0 | 0 |
| Aspartate aminotransferase increased | 1 (3.3) | 0 | 0 |
| Blood glucose increased | 0 | 0 | 1 (2.5) |
| Transaminases increased | 1 (3.3) | 0 | 0 |
| Nervous system disorders |  |  |  |
| Total patients with ≥1 AE | 2 (6.7) | 2 (6.7) | 1 (2.5) |
| Total number of AEs | 2 | 2 | 1 |
| Headache | 1 (3.3) | 1 (3.3) | 0 |
| Presyncope | 1 (3.3) | 0 | 1 (2.5) |
| Dizziness | 0 | 1 (3.3) | 0 |
| Infections and infestations |  |  |  |
| Total patients with ≥1 AE | 1 (3.3) | 1 (3.3) | 2 (5.0) |
| Total number of AEs | 2 | 1 | 2 |
| COVID-19 pneumonia | 1 (3.3) | 1 (3.3) | 0 |
| COVID-19 | 0 | 0 | 1 (2.5) |
| Lower respiratory tract infection | 0 | 0 | 1 (2.5) |
| Urinary tract infection | 1 (3.3) | 0 | 0 |
| Blood and lymphatic system disorders |  |  |  |
| Total patients with ≥1 AE | 1 (3.3) | 2 (6.7) | 0 |
| Total number of AEs | 1 | 2 | 0 |
| Anemia | 0 | 1 (3.3) | 0 |
| Hypochromic anemia | 1 (3.3) | 0 | 0 |
| Neutropenia | 0 | 1 (3.3) | 0 |
| Injury, poisoning, and procedural complications |  |  |  |
| Total patients with ≥1 AE | 0 | 0 | 3 (7.5) |
| Total number of AEs | 0 | 0 | 3 |
| Accidental overdose | 0 | 0 | 1 (2.5) |
| Medication error | 0 | 0 | 1 (2.5) |
| Skin laceration | 0 | 0 | 1 (2.5) |
| Cardiac disorders |  |  |  |
| Total patients with ≥1 AE | 1 (3.3) | 0 | 1 (2.5) |
| Total number of AEs | 1 | 0 | 1 |
| Atrial flutter | 0 | 0 | 1 (2.5) |
| Bradycardia | 1 (3.3) | 0 | 0 |
| Musculoskeletal and connective tissue disorders |  |  |  |
| Total patients with ≥1 AE | 2 (6.7) | 0 | 0 |
| Total number of AEs | 2 | 0 | 0 |
| Arthralgia | 1 (3.3) | 0 | 0 |
| Pain in extremity | 1 (3.3) | 0 | 0 |
| Respiratory, thoracic, and mediastinal disorders |  |  |  |
| Total patients with ≥1 AE | 1 (3.3) | 1 (3.3) | 0 |
| Total number of AEs | 1 | 2 | 0 |
| Cough | 0 | 1 (3.3) | 0 |
| Dyspnea | 1 (3.3) | 0 | 0 |
| Oropharyngeal pain | 0 | 1 (3.3) | 0 |
| Skin and subcutaneous tissue disorders |  |  |  |
| Total patients with ≥1 AE | 1 (3.3) | 1 (3.3) | 0 |
| Total number of AEs | 1 | 1 | 0 |
| Eczema | 0 | 1 (3.3) | 0 |
| Urticaria | 1 (3.3) | 0 | 0 |
| Ear and labyrinth disorders |  |  |  |
| Total patients with ≥1 AE | 0 | 1 (3.3) | 0 |
| Total number of AEs | 0 | 1 | 0 |
| Tinnitus | 0 | 1 (3.3) | 0 |
| Metabolism and nutrition disorders |  |  |  |
| Total patients with ≥1 AE | 1 (3.3) | 0 | 0 |
| Total number of AEs | 1 | 0 | 0 |
| Hypokalemia | 1 (3.3) | 0 | 0 |
| Vascular disorders |  |  |  |
| Total patients with ≥1 AE | 1 (3.3) | 0 | 0 |
| Total number of AEs | 1 | 0 | 0 |
| Hypotension | 1 (3.3) | 0 | 0 |

Abbreviation: AE, adverse event.

**Supplementary Table 8. Adverse Events Leading to Treatment Discontinuation (Safety Population)**

|  | Bemnifosbuvir 550 mg  (*n* = 30) | Bemnifosbuvir 1,100 mg  (*n* = 30) | Pooled Placebo  (*n* = 40) |
| --- | --- | --- | --- |
| Patients with ≥1 AE | 0 | 5 (16.7%) | 1 (2.5%) |
| Total number of AEs | 0 | 7 | 3 |
| Gastrointestinal disorders |  |  |  |
| Total number of patients with ≥1 AE | 0 | 5 (16.7%) | 1 (2.5%) |
| Total number of AEs | 0 | 7 | 2 |
| Vomiting | 0 | 5 (16.7%) | 1 (2.5%) |
| Nausea | 0 | 2 (6.7%) | 1 (2.5%) |
| General disorders and administration site conditions |  |  |  |
| Total number of patients with ≥1 AE | 0 | 0 | 1 (2.5%) |
| Total number of AEs | 0 | 0 | 1 |
| Mass | 0 | 0 | 1 (2.5%) |

Abbreviation: AE, adverse event.

**Supplementary Figure 1.**


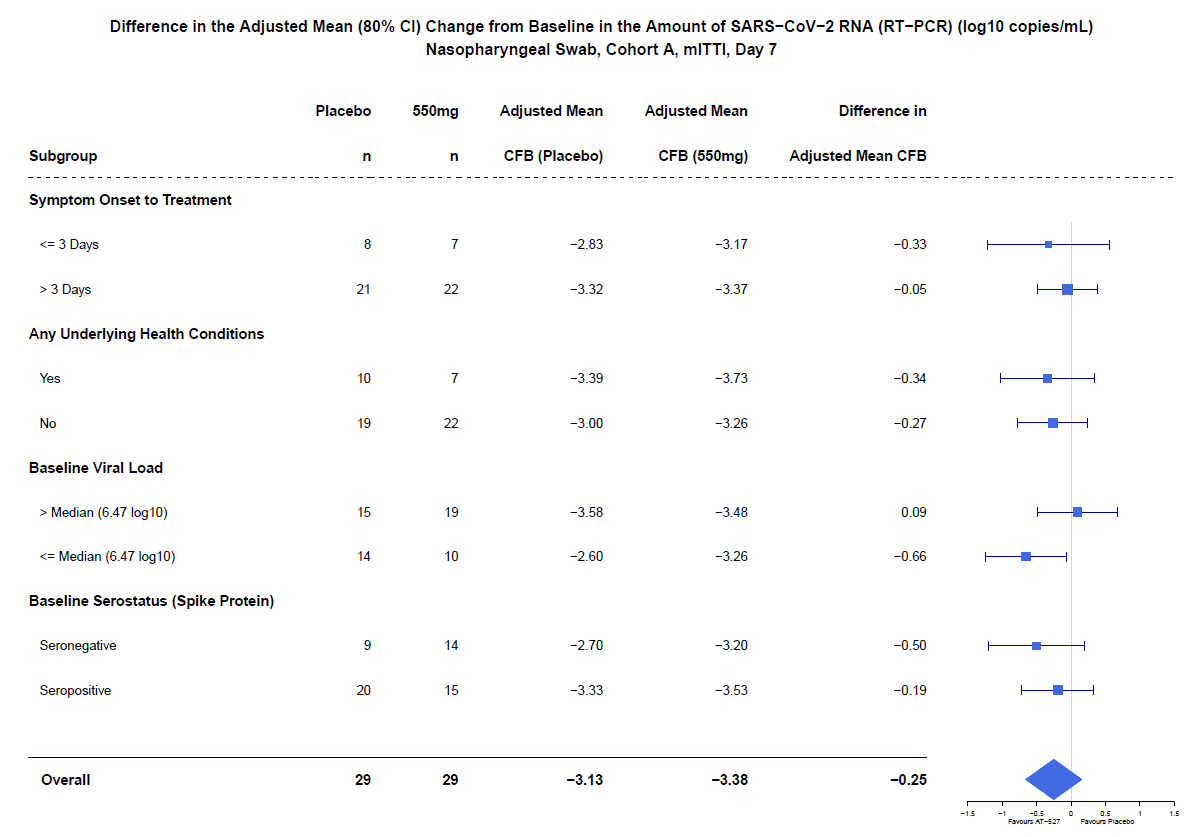
A)


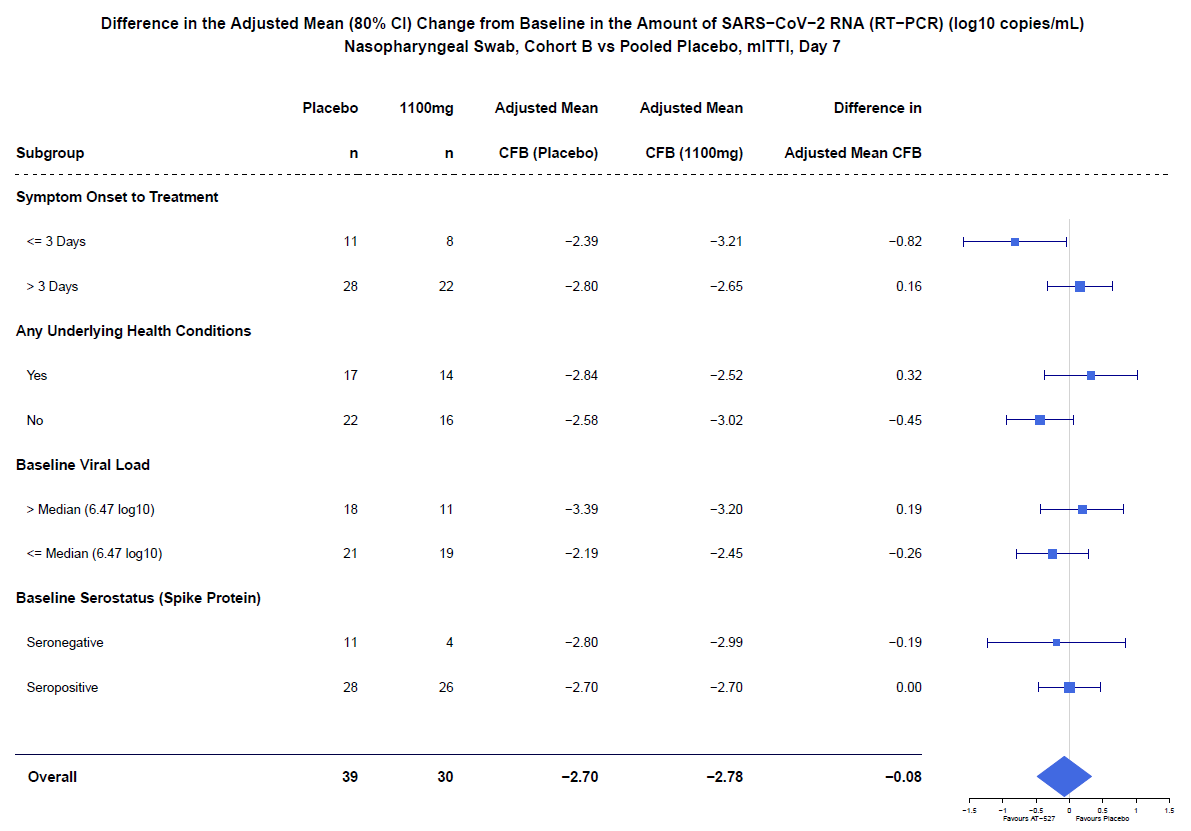
B)

Adjusted mean change from baseline in amount of SARS-CoV-2 virus RNA by RT-PCR at day 7 in key subgroups forest plot; A) Cohort A vs Cohort A placebo; B) Cohort B vs pooled placebo.

AT-527 also known as bemnifosbuvir.

ANCOVA, analysis of covariance; CFB, change from baseline; RNA, ribonucleic acid; RT-PCR, reverse transcription polymerase chain reaction; SARS-CoV-2, Severe Acute Respiratory Syndrome Coronavirus 2.

**Supplementary Figure 2.**


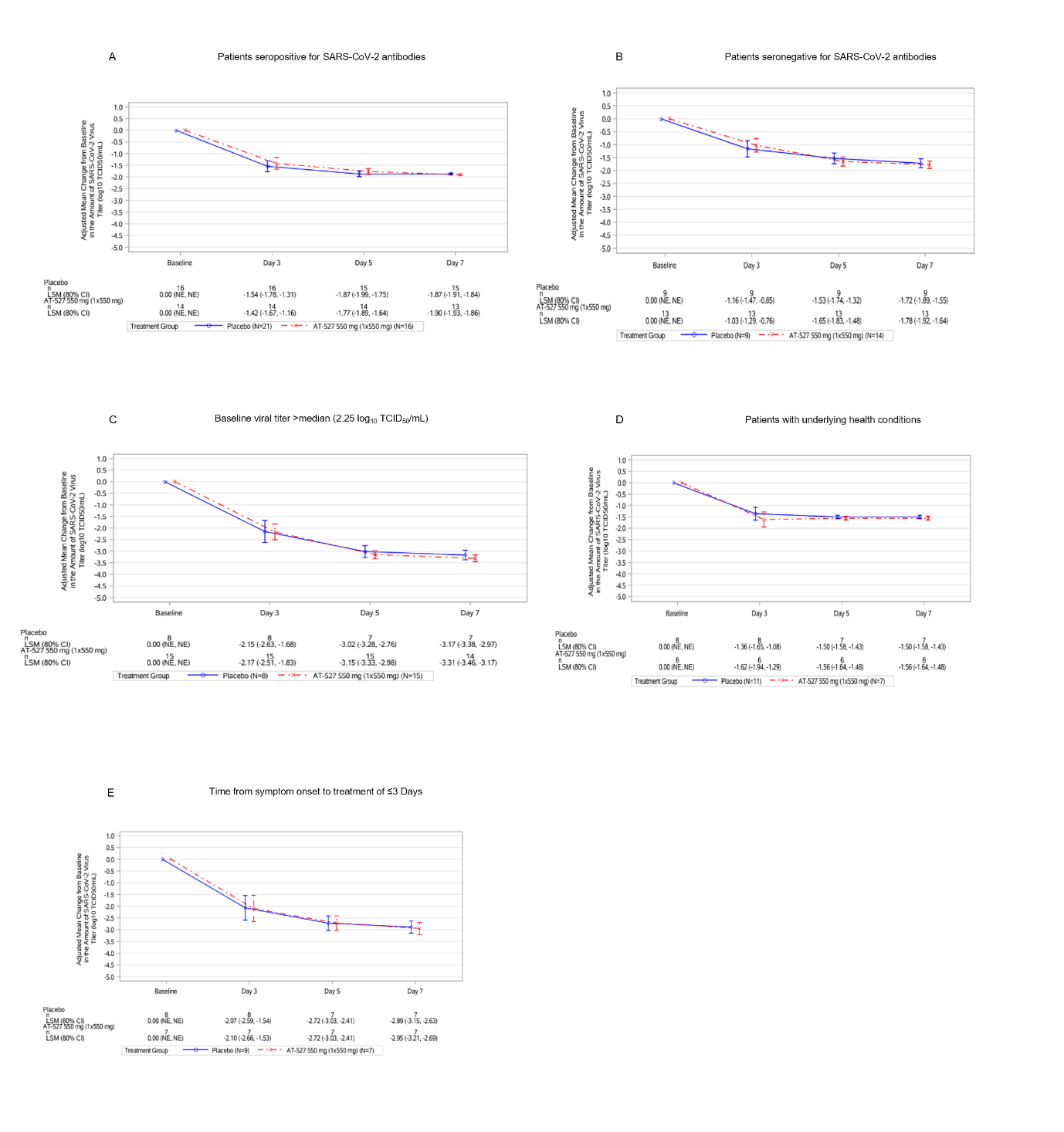


Exploratory analysis: adjusted change from baseline in amount of SARS-CoV-2 infectious virus titer at specified timepoints in key subgroups (Cohort A vs Cohort A placebo).

AT-527 also known as bemnifosbuvir. Only patients that had at least one positive test result during the study were included.

CI, confidence interval; LSM, least squares mean; NE, not evaluable; SARS-CoV-2, Severe Acute Respiratory Syndrome Coronavirus 2; TCID_50_, 50% tissue culture infectious dose.

**Supplementary Figure 3.**


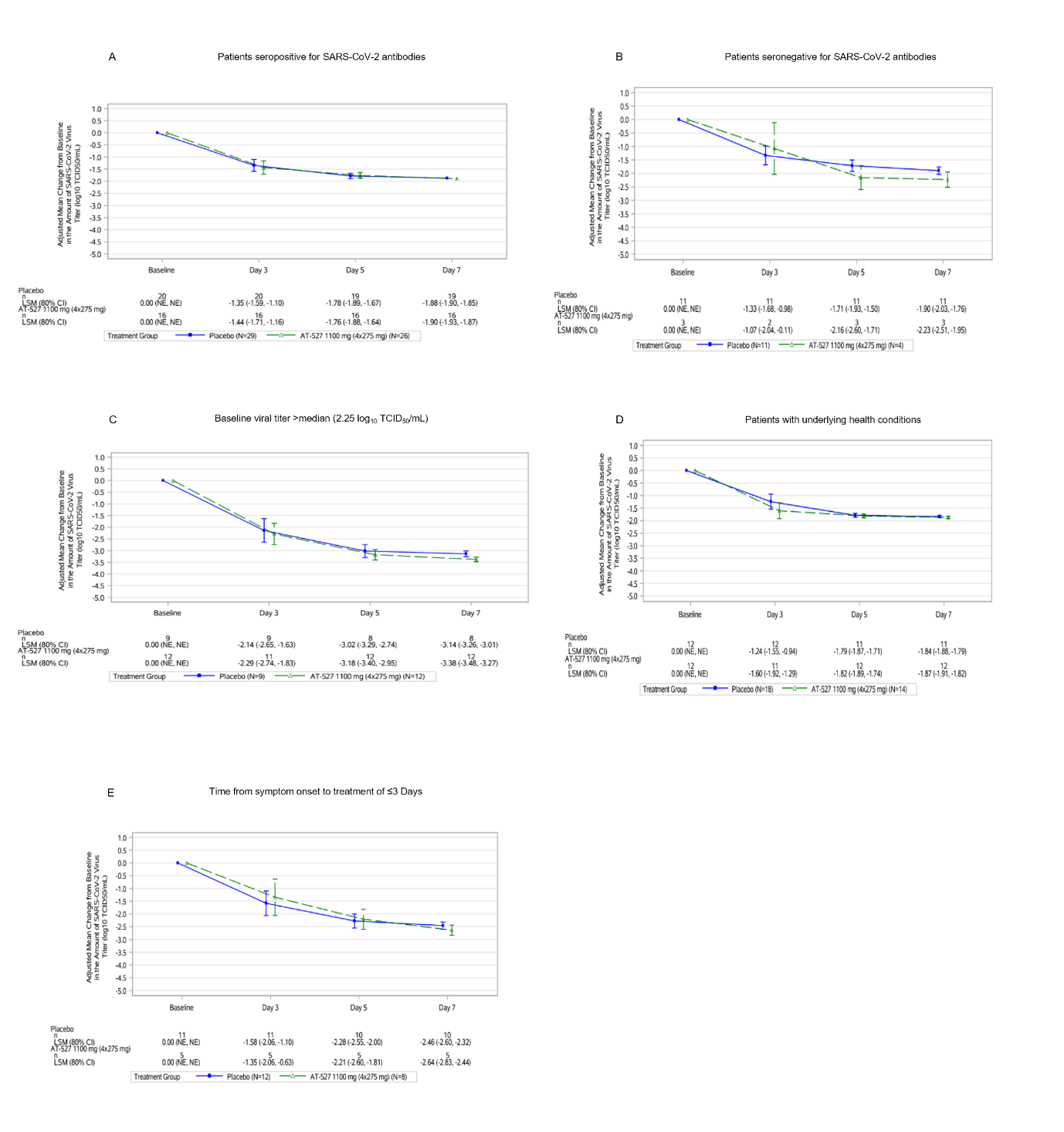


Exploratory analysis: adjusted change from baseline in amount of SARS-CoV-2 infectious virus titer at specified timepoints in key subgroups (Cohort B vs pooled placebo).

AT-527 also known as bemnifosbuvir. Only patients that had at least one positive test result during the study were included.

CI, confidence interval; LSM, least squares mean; NE, not evaluable; SARS-CoV-2, Severe Acute Respiratory Syndrome Coronavirus 2; TCID_50_, 50% tissue culture infectious dose.

**Supplementary Figure 4.**


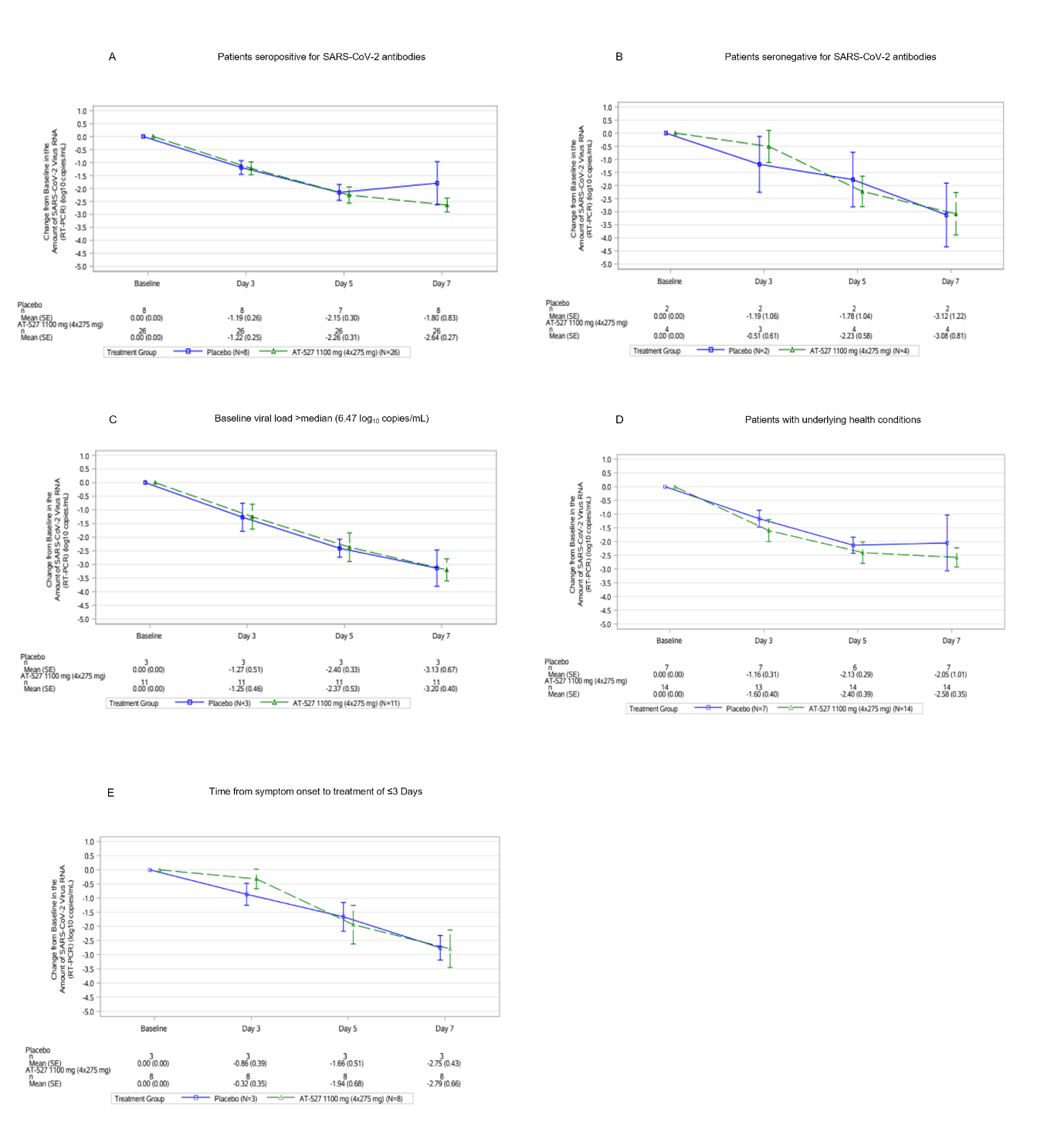


Change from baseline in amount of SARS-CoV-2 virus RNA by RT-PCR at specified timepoints in key subgroups (Cohort B vs Cohort B placebo).

AT-527 also known as bemnifosbuvir.

RNA, ribonucleic acid; RT-PCR, reverse transcription polymerase chain reaction; SARS-CoV-2, Severe Acute Respiratory Syndrome Coronavirus 2; SE, standard error.

**Supplementary Figure 5.**


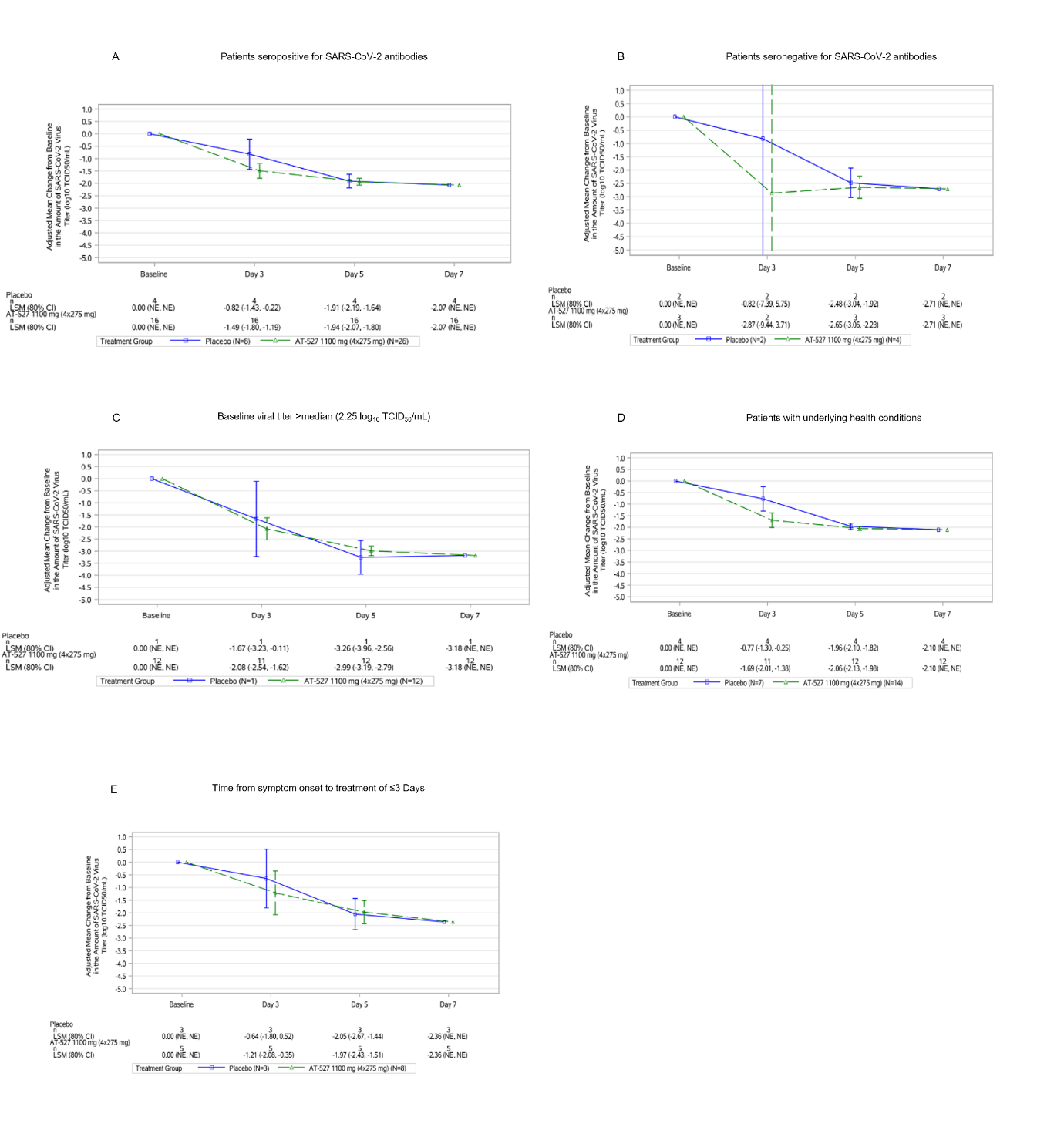


Adjusted change from baseline in amount of SARS-CoV-2 infectious virus titer at specified timepoints plot in key subgroups (Cohort B vs Cohort B placebo).

AT-527 also known as bemnifosbuvir. Only patients that had at least one positive test result during the study were included.

CI, confidence interval; LSM, least squares mean; NE, not evaluable; SARS-CoV-2, Severe Acute Respiratory Syndrome Coronavirus 2; TCID_50_, 50% tissue culture infectious dose

**Supplementary Figure 6.**


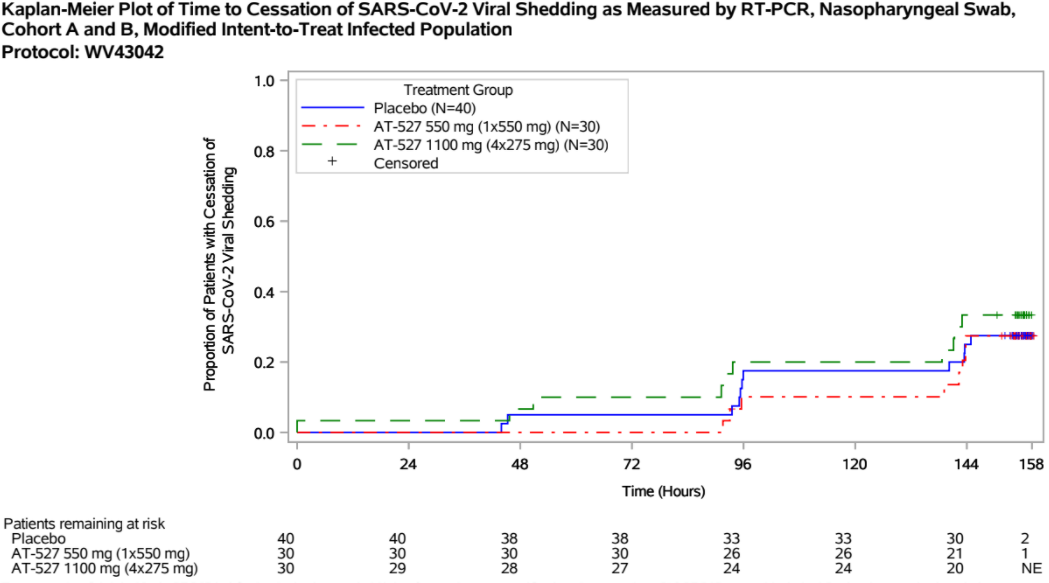


Time to cessation of SARS-CoV-2 viral RNA shedding by RT-PCR (Cohort A and B vs pooled placebo).

AT-527 also known as bemnifosbuvir.

NE, not evaluable; RNA, ribonucleic acid; RT-PCR, reverse transcription polymerase chain reaction; SARS-CoV-2, Severe Acute Respiratory Syndrome Coronavirus 2.

**Supplementary Figure 7.**


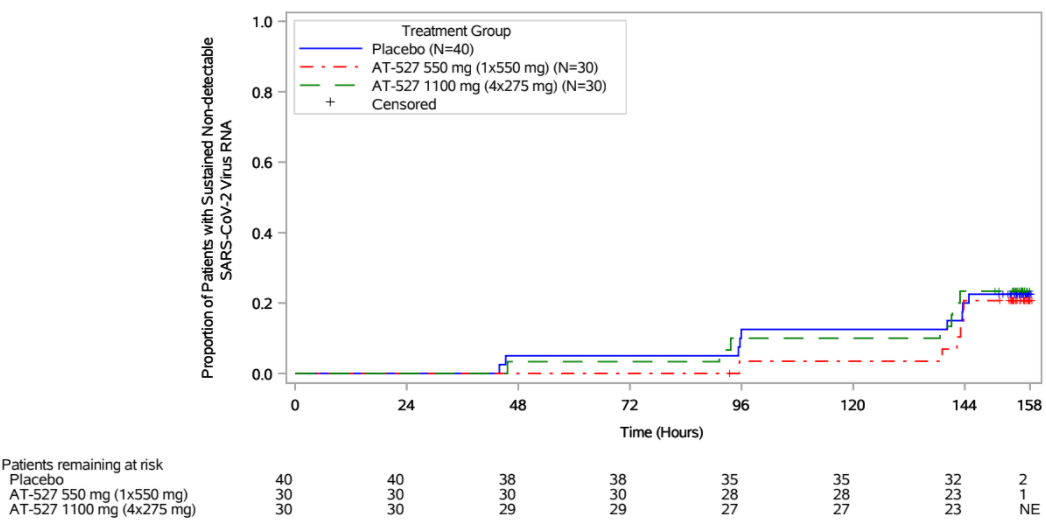


Time to sustained non-detectable nasopharyngeal SARS-CoV-2 virus RNA by RT-PCR (Cohort A and B vs pooled placebo).

AT-527 also known as bemnifosbuvir.

NE, not evaluable; RNA, ribonucleic acid; RT-PCR, quantitative reverse transcription polymerase chain reaction; SARS-CoV-2, Severe Acute Respiratory Syndrome Coronavirus 2.

**Supplementary Figure 8.**


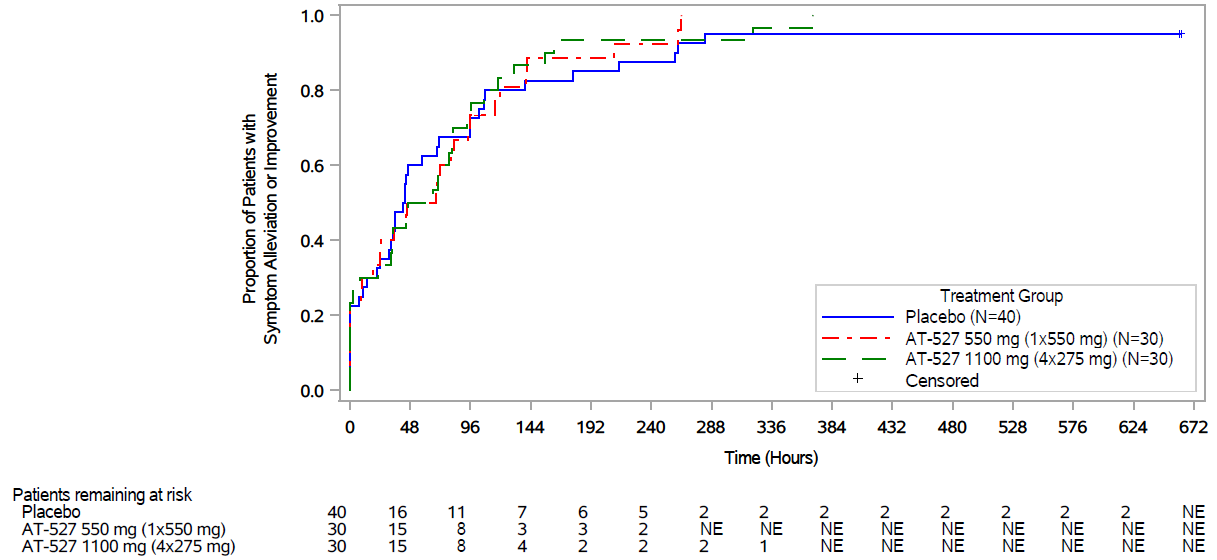


Time to alleviation or improvement of COVID-19 symptoms maintained for 21.5 hours (Cohort A and B vs pooled placebo).

AT-527 also known as bemnifosbuvir.

COVID-19, coronavirus disease 2019; NE, not evaluable.

**Supplementary Figure 9.**

**A.**


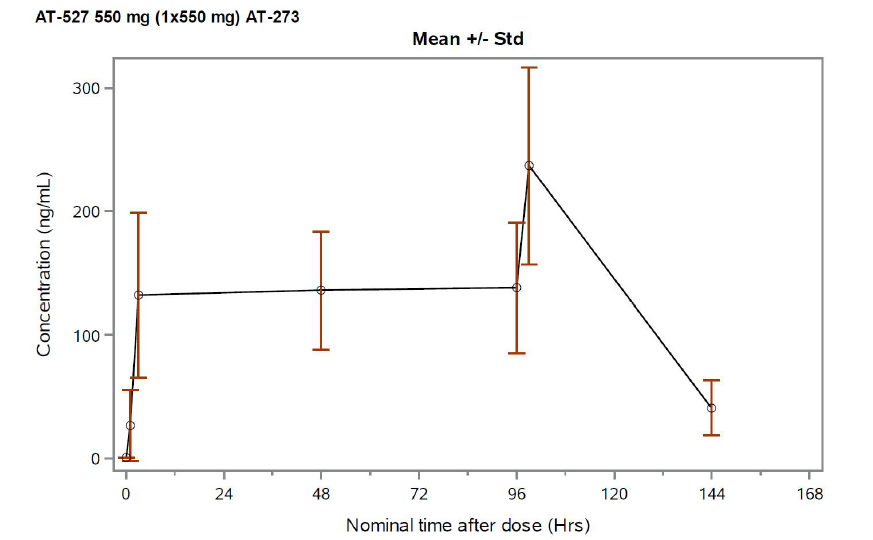


**B.**


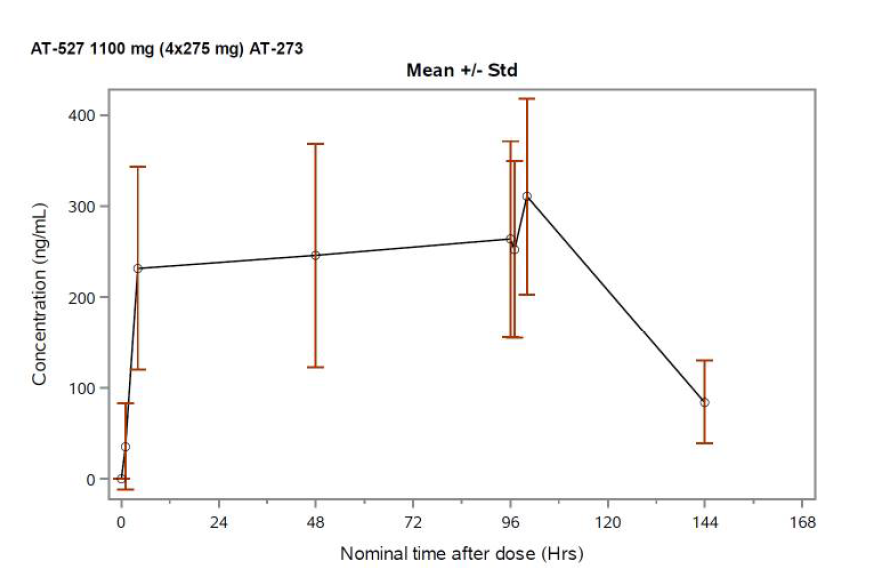


Mean (+SD) concentrations of AT-273 in plasma versus nominal time by treatment group; A) Cohort A; B) Cohort B.

Hrs, hours; SD, standard deviation.

**Supplementary Figure 10.**


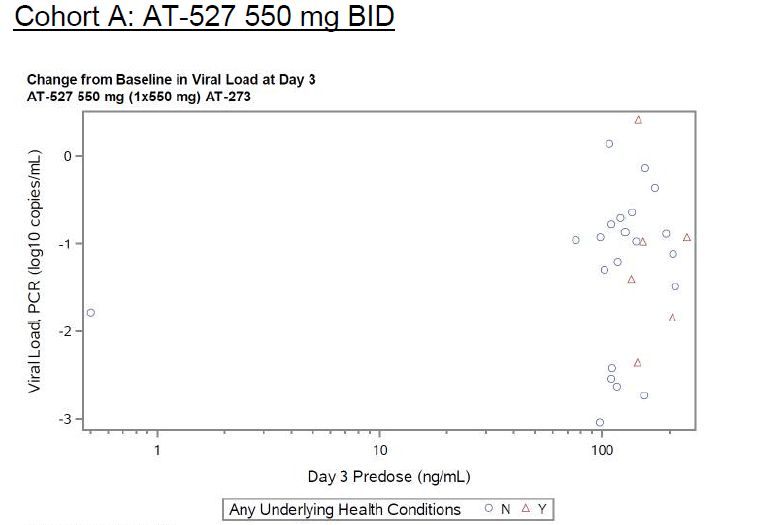
**A.**

**
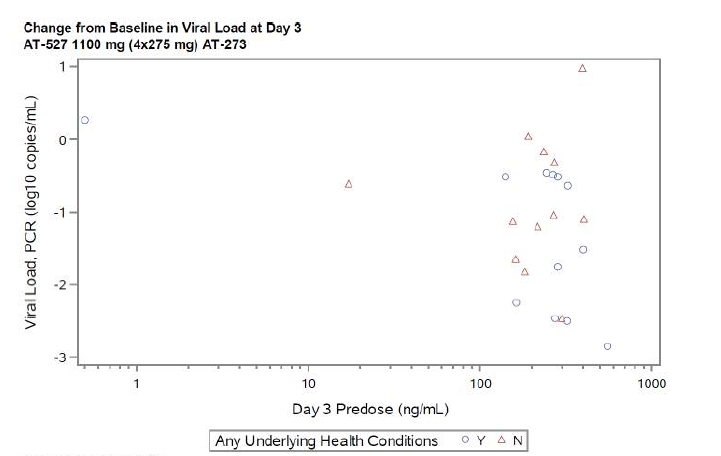
B.**

Scatter plot of change from baseline in SARS-CoV-2 virus RNA by RT-PCR at day 3 versus plasma AT-273 trough concentration at day 3 (log-scaled) by treatment group; A) Cohort A; B) Cohort B.

PCR, polymerase chain reaction; RNA, ribonucleic acid; RT-PCR, quantitative reverse transcription polymerase chain reaction; SARS-CoV-2, Severe Acute Respiratory Syndrome Coronavirus 2.

**Supplementary Figure 11.**

**A.**


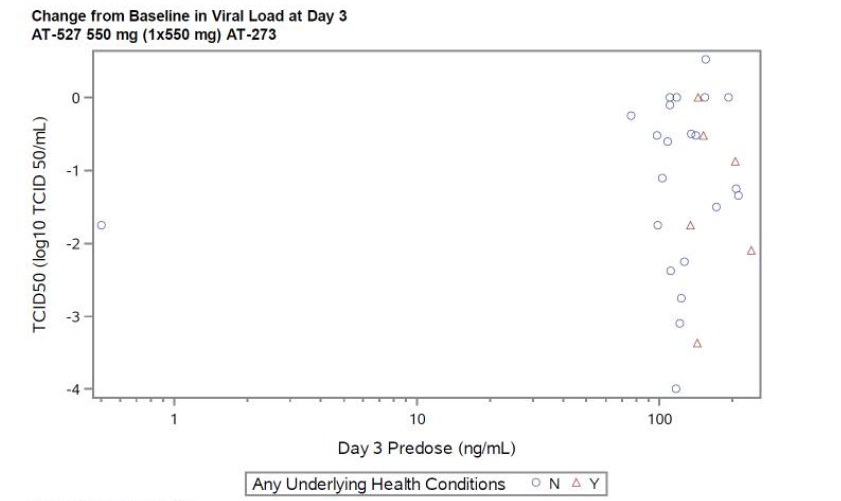


**B.**


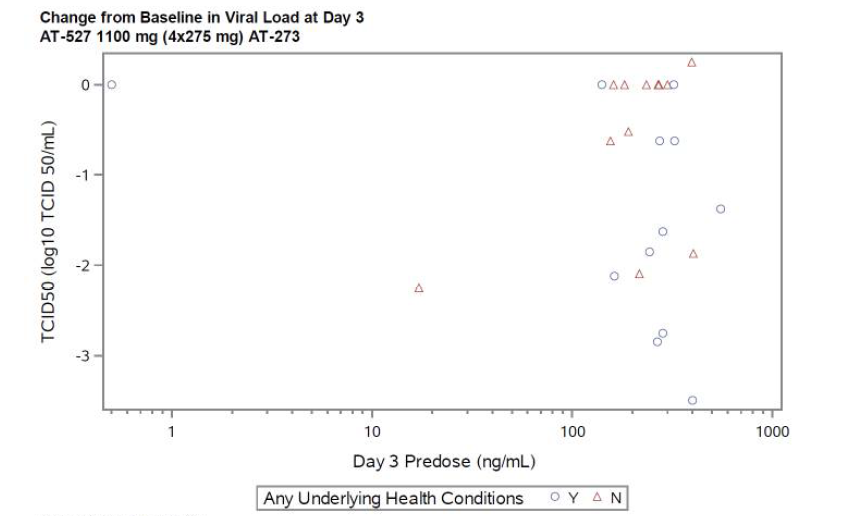


Scatter plot of change from baseline in infectious virus titer at day 3 versus plasma AT-273 trough concentration at day 3 (log-scaled) by treatment group; A) Cohort A; B) Cohort B.

TCID50, 50% tissue culture infectious dose.

**Supplementary Figure 12.**


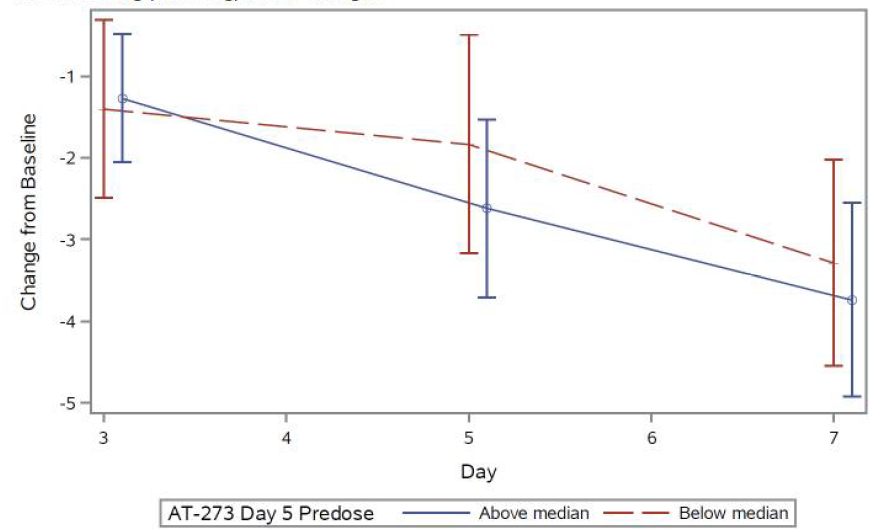
 **A.**


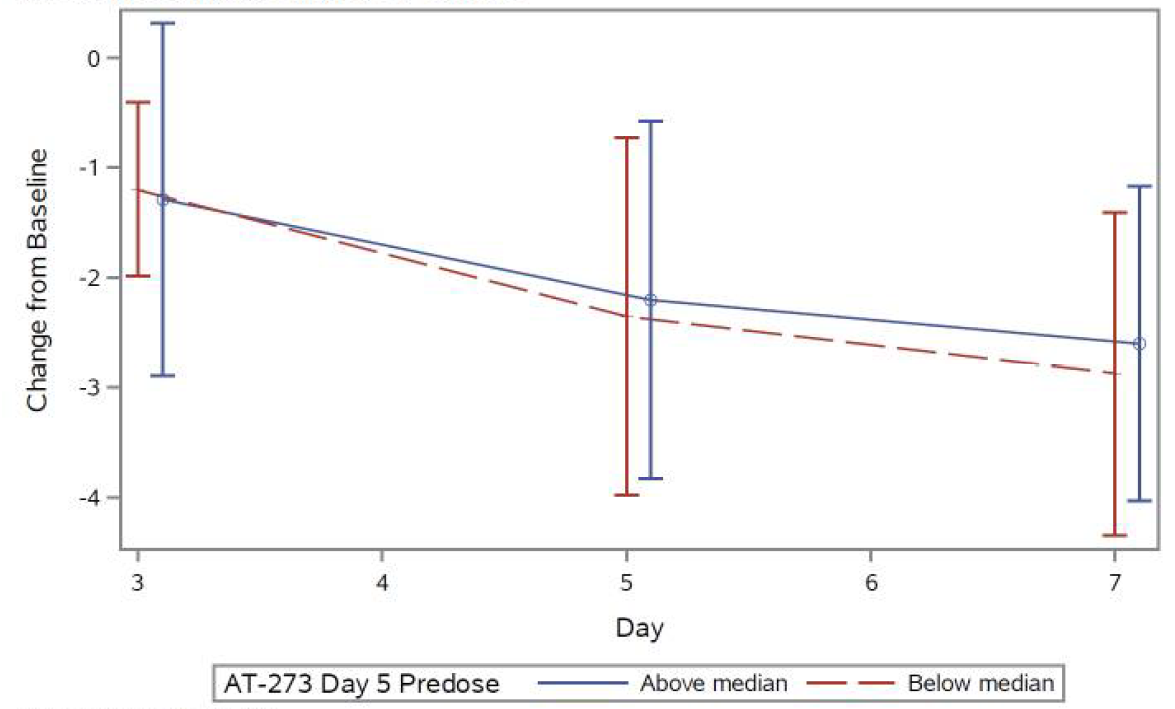
**B.**

Mean (SD) change from baseline in SARS-CoV-2 virus RNA by RT-PCR by treatment group versus nominal time: trough AT-273 concentration in plasma at day 5 above or below median value; A) Cohort A; B) Cohort B.

RNA, ribonucleic acid; RT-PCR, quantitative reverse transcription polymerase chain reaction; SARS-CoV-2, Severe Acute Respiratory Syndrome Coronavirus 2; SD, standard deviation.
